# Avatar-Based Patient Monitoring With Peripheral Vision: A Multicenter Comparative Eye-Tracking Study

**DOI:** 10.2196/13041

**Published:** 2019-07-17

**Authors:** Juliane Pfarr, Michael T Ganter, Donat R Spahn, Christoph B Noethiger, David W Tscholl

**Affiliations:** 1 Institute of Anesthesiology University and University Hospital Zurich Zurich Switzerland; 2 Institute of Anesthesiology Kantonsspital Winterthur Winterthur Switzerland

**Keywords:** anesthesia, critical care, computers, diagnosis, patient monitoring, situation awareness, perception, vision

## Abstract

**Background:**

Continuous patient monitoring has been described by the World Health Organization as extremely important and is widely used in anesthesia, intensive care medicine, and emergency medicine. However, current state-of-the-art number- and waveform-based monitoring does not ideally support human users in acquiring quick, confident interpretations with low cognitive effort, and there are additional problematic aspects such as alarm fatigue. We developed a visualization technology (Visual Patient), specifically designed to help caregivers gain situation awareness quickly, which presents vital sign information in the form of an animated avatar of the monitored patient. We suspected that because of the way it displays the information as large, colorful, moving graphic objects, caregivers might be able to perform patient monitoring using their peripheral vision, which may facilitate quicker detection of anomalies, independently of acoustic alarms.

**Objective:**

In this study, we tested the hypothesis that avatar-based monitoring, when observed with peripheral vision only, increases the number of perceptible changes in patient status as well as caregivers’ perceived diagnostic confidence compared with a high-fidelity simulation of conventional monitoring, when observed with peripheral vision only.

**Methods:**

We conducted a multicenter comparative study with a within-participant design in which anesthesiologists with their peripheral field of vision looked at 2 patient-monitoring scenarios and tried to identify changes in patient status. To ensure the best possible experimental conditions, we used an eye tracker, which recorded the eye movements of the participants and confirmed that they only looked at the monitoring scenarios with their peripheral vision.

**Results:**

Overall, 30 participants evaluated 18 different patient status changes with each technology (avatar and conventional patient monitoring). With conventional patient monitoring, participants could only detect those 3 changes in patient status that are associated with a change in the auditory pulse tone display, that is, tachycardia (faster beeping), bradycardia (slower beeping), and desaturation (lower pitch of beeping). With the avatar, the median number of detected vital sign changes quadrupled from 3 to 12 (*P*<.001) in scenario 1, and more than doubled from 3 to 8 (*P*<.001) in scenario 2. Median perceived diagnostic confidence was *confident* for both scenarios with the avatar and *unconfident* in scenario 1 (*P*<.001), and *very unconfident* in scenario 2 (*P=*.024) with conventional monitoring.

**Conclusions:**

This study introduces the concept of peripheral vision monitoring. The test performed showed clearly that an avatar-based display is superior to a standard numeric display for peripheral vision. Avatar-based monitoring could potentially make much more of the patient monitoring information available to caregivers for longer time periods per case. Our results indicate that the optimal information transmission would consist of a combination of auditory and avatar-based monitoring.

## Introduction

### Patient Monitoring Background

In its Guidelines for Safe Surgery, the World Health Organization describes continuous patient monitoring by an attentive and professionally trained caregiver as *extremely important* for perioperative safety [1]. Noninvasive standard monitoring offers an excellent risk-benefit ratio, as it is not dangerous for patients, yet through earlier, clearer detection of vital sign abnormalities than is possible by assessing clinical signs alone, it may prevent potential catastrophic complications, for example, brain damage [2-6]. Patient monitoring enjoys widespread acceptance among caregivers and professional associations in anesthesia, intensive care medicine, and emergency medicine [1,7]. With technological progress in sensor and computer technology, patient monitoring can be expected to increasingly be extended to areas where patients are currently not routinely monitored, thereby detecting vital sign anomalies even earlier than is now the case, for example, in general hospital wards [8].

### Introduction to Conventional Patient Monitoring

Human factor experts have long recognized that representation of vital sign data in the form of a multitude of numbers and waveforms in today’s state-of-the-art monitors does not ideally support human users in arriving at a quick interpretation with a high degree of confidence and with a low cognitive effort [9]. Several characteristic aspects of conventional representation are responsible for this: (1) people can only read numbers one by one [10]; (2) the numbers displayed represent low-level data and, only indirectly, the relevant information [11]; (3) many of the numbers displayed have the same ranges, for example, pulse rate, blood pressure, oxygen saturation, and others can all be *95*; (4) people can only remember 7 digits plus or minus 2 at a time in their short-term memory [12]. The resulting need for piecemeal data acquisition, mental decoding, and subsequent interpretation of the meaning of the data requires much time and cognitive effort on the part of the caregiver to obtain adequate situation awareness of the patient’s current condition. Situation awareness refers to the correct perception of a situation and its expected course [13]. It is an essential prerequisite for informed decision making, and research has identified situation awareness errors in up to 80% of adverse events [14,15]. Patient monitors, to mitigate some of their limitations, use audible and visual alarms to warn caregivers when vital signs diverge from their normal range. However, around 80% of issued alarms are false-positives that do not lead to a therapeutic consequence, leading to a *crying wolf phenomenon*, that is, caregivers experiencing alarm fatigue, with resulting failure to detect truly positive alarms [16]. In a recent study, 56% (14/25) of anesthesiologists agreed with the statement that problems with alarm settings make their work with patient monitors more difficult [17]. Studies investigating patient monitoring behavior have found that anesthesia providers look at patient monitors for only about 5% of the time during a procedure and that they tend to look less often in high-workload situations, when other tasks cause cognitive saturation [18,19].

### Introduction to Avatar-Based Monitoring

One possible way to optimize the process of information transfer between patient monitors and caregivers may be to present the vital sign information in the form of graphical objects [9,20,21]. Applying principles of situation awareness design [22], we developed an avatar-based visualization technology (Visual Patient) that presents vital sign information as an animated avatar of the monitored patient and is specifically aimed at helping caregivers gain situation awareness quickly and with low cognitive effort. In a previous study [23], we found that compared with conventional technology monitoring the same short clinical scenarios, this technology increased both the number of correctly perceived vital signs and the diagnostic confidence reported by the participating anesthesia providers, while reducing perceived workload. Furthermore, users considered the technology intuitive, easy to learn, and helpful [24].

### Patient Monitoring With Peripheral Vision

**Figure 1:**
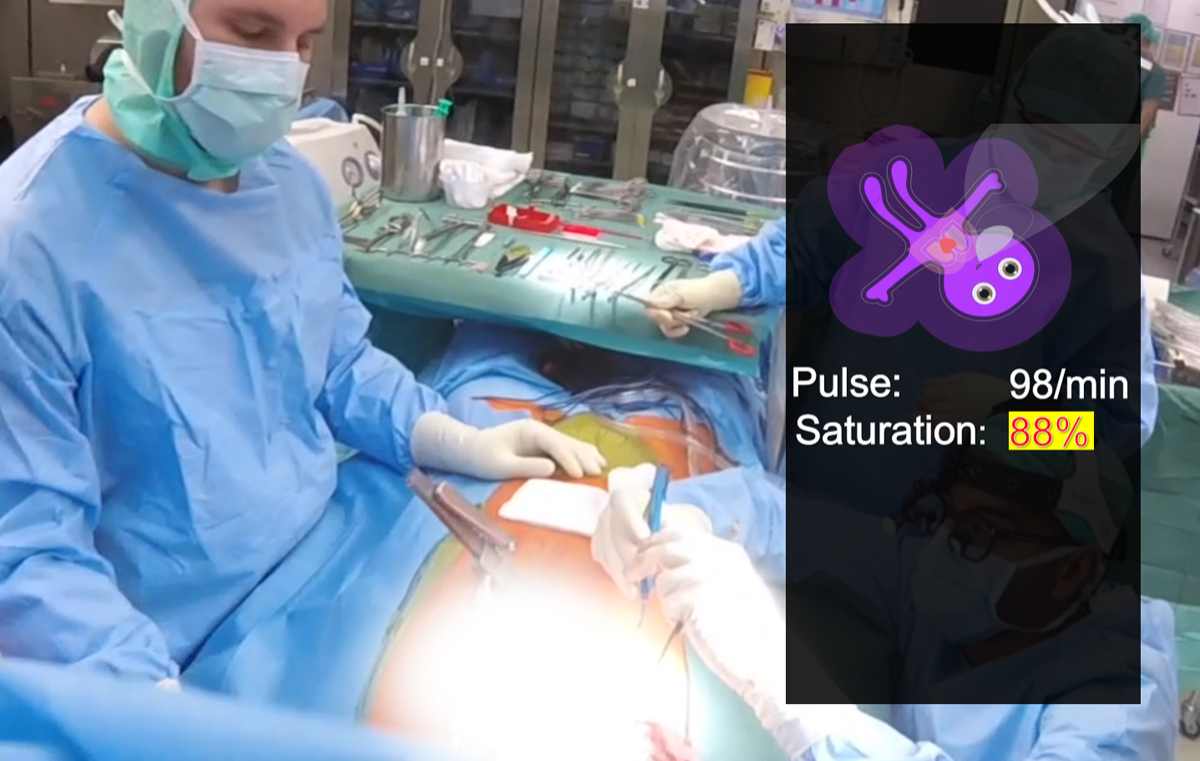
An example of a possible future application of peripheral vision monitoring in the form of an augmented reality application for patient monitoring, as Philips (Koninklijke Philips NV, Amsterdam, Netherlands) has tested on a Google (Alphabet Inc) Glass headset. If the reader looks at the center of the operating field in this photo, they can no longer read the numerical monitoring information, for example, saturation: 88%, however, they can still see that the avatar is purple and thus desaturated.

We suspected that because of the way the avatar representation displays the information as large, colorful, moving graphical objects, caregivers might be able to perform patient monitoring using their peripheral vision. Conventional monitoring is particularly unsuitable for monitoring with peripheral vision because of the presentation of information in the form of numbers and figures as described above. To be able to read a number, a caregiver must fix their foveal or sharp vision directly on the number they intend to read. Foveal vision corresponds to the small central part of the retina in which a large number of cones are concentrated. Outside the area of foveal vision, color perception deteriorates and vision becomes blurry, rendering people unable to read glyphs with their peripheral vision [10,25,26]. Patient monitoring with peripheral vision could provide several theoretical advantages. It could increase the time per case that a caregiver has direct visual contact with the monitoring information, from approximately 5% of the time during which they observe the monitor with foveal vision to all the time they have the monitor in their peripheral visual field. Considering that the human binocular visual field encompasses approximately 214 arc degrees horizontally and 150 arc degrees vertically, the monitor is within the visual field virtually all of the time [27]. Peripheral vision monitoring may facilitate quicker detection of anomalies independently of acoustic alarms. Furthermore, the feeling of always keeping an eye on the situation may reduce caregiver stress levels, as uncertainty is a psychological stress factor [22,28]. Figure 1 shows an example of a potential future use of peripheral vision monitoring.

### Objective

In this study, we tested the hypothesis that avatar-based monitoring with peripheral vision increases the number of vital signs perceptible as well as perceived diagnostic confidence compared with conventional monitoring with peripheral vision.

## Methods

The Cantonal Ethics Committee in Zurich, Switzerland, reviewed the protocol of this study and issued a declaration of no objection (Business Administration System for Ethical Committees-Number 2017-00795 issued on October 23, 2017). All participants gave their written informed consent to the use of the data collected for scientific evaluation. The participants participated voluntarily in this study and received no financial compensation.

### Description of Visual Patient Technology

The version of the technology used in this study can display the 11 most commonly monitored vital signs: pulse rate, blood pressure, oxygen saturation, ST segment of the electrocardiogram, central venous pressure, respiratory rate, tidal volume, expiratory carbon dioxide concentration, body temperature, brain activity, and degree of neuromuscular relaxation.

We developed the avatar as a situation-awareness tool, analogous to the so-called synthetic vision technology in aviation, and according to the principles of situation-awareness design and logic [22,29]. The synthetic vision technology renders a virtual image of the environment from data measured by the aircraft, for example, altitude information, and global positioning system–referenced elevation data. To the pilot, the virtual image it creates looks identical to the view outside the window in perfect weather. This similarity is what makes it intuitively understandable and allows for the quick and uncomplicated perception of the flight situation. Visual Patient technology does the same, in this case, by creating a virtual image of the patient from vital sign data. Similar to synthetic vision technology, it presents the numerical data in a way that corresponds to the physical phenomena they engender in the patient. For example, low oxygen saturation is represented with cyanotic skin color because this is what hypoxia causes in a patient, and that is what caregivers expect.

This so-called direct presentation of information eliminates the necessity for a caregiver to calculate the relevant information mentally from lower level data, for example, is the patient hypoxic or not if oxygen saturation is 85% [11]. In addition to this direct presentation of information, the 2 other main characteristics of the avatar are the preprocessing of the data for each vital sign into the categories *too low*, *normal*, or *too high* and the presentation of the vital sign information in several visualizations at the same time. For example, caregivers can judge the respiratory rate by the respiratory rate of the avatar’s lungs as well as the formation rate of the carbon dioxide cloud exhaled by the avatar.

These functions combined translate the multitude of numerical values into an animated model of the patient situation, which the caregiver can evaluate and remember at a glance. The translation of the vital signs into the avatar model takes place in real time from the monitoring data. We have described the validation and evaluation process of the avatar in detail in previous studies [23,24].

### Study Participants

For this study, we included anesthesia providers in 2 study centers. The University Hospital of Zurich, a University maximum care hospital with more than 30,000 anesthesia cases per year and the Cantonal Hospital of Winterthur, a regional teaching hospital with more than 10,000 cases per year. Both centers included balanced proportions of female and male participants as well as equal proportions of the different professional groups (senior and resident physicians and anesthesia nurses). To participate in the study, we freed the participants from their respective tasks during their regular working hours so that they could participate undisturbed by external influences.

### Study Procedure

We collected the data for this experiment as part of a session in which we also collected the data for 2 more experiments. Each of the participants sat in a quiet room of the University Hospital Zurich or the Cantonal Hospital Winterthur accompanied by a data collector, who guided the participant through the experiments. During the experiments, the participants sat in front of 2 computer screens. Initially, they watched an instructional video about Visual Patient technology and familiarized themselves with the layout of the conventional monitoring display. After the introduction and after they had completed a short personal information questionnaire (gender, age, and years of professional anesthesia experience), the experiments were conducted in sequence. In a pilot study, we discovered that the approximate duration of 1 data collection session would be about 1 hour and 15 min. With 2 short pauses between the 3 experiments, we considered this duration acceptable for the participants’ ability to remain concentrated during the tests. The peripheral vision experiment conducted for this study was experiment number 3. We will report on the results of experiments 1 and 2 in separate papers. For all 3 experiments, we used an iPad-based (Apple Inc) data entry tool for data entry during the experiments [30].

### Peripheral Vision Experiment

As the first step in the peripheral vision experiment, we positioned the participants at a distance of approximately 60 cm directly in front of a laptop screen. Then a stationary eye tracker (Gazepoint GP3 by Gazepoint) was calibrated to capture the foveal vision, that is, gaze plot, a sequence and durations of visual fixations, of the participants on the laptop screen directly in front of them.

For the peripheral vision scenario, we played the monitoring scenario evaluated by the participants on the second screen on the left side of the participant, which we placed at an angle of 45° to the visual axis of the participants and, thus, in their peripheral field of view. We instructed the participants never to look away from the central screen during the entire 8-min test. On this central screen, we showed a Microsoft (Microsoft Corp) PowerPoint presentation showing an animated graphic of a cat in an endless loop. This ensured that the foveal vision of the participants remained on the central screen and that they, therefore, could only see the scenario played on the second monitor with their peripheral field of vision. This method ensured that the volunteers really only looked at the scenario with their peripheral vision and that the data collected were, therefore, valid for this evaluation. Even if the participant’s view were to wander to the left edge of the screen, the monitor at a 45° angle, that is, the monitor on which the monitoring scenario was running, would still be deep in the middle peripheral field of view, which extends from 30° to 60° from the point of sharpest vision.

We used this method based on research that showed that the human observer’s vision is only sharp enough to read numbers or glyphs at an angle of 10° around the point of sharp vision. At a distance of 60 cm from the screen, the area of sharp vision is about the size of a fingernail, or more precisely, a circle about 2 cm in diameter. Outside of this small area, people only recognize blurred images and monochromatic colors [10,25]. In this study, we only included participants who did not look to the left of the monitor more than twice during the test. Two short glances at the monitor would, if both glances were successful and allowed for perception of 2 status changes, only account for a maximum of 5% of the participant’s dataset, given the 36 evaluated status changes per dataset.

**Figure 2:**
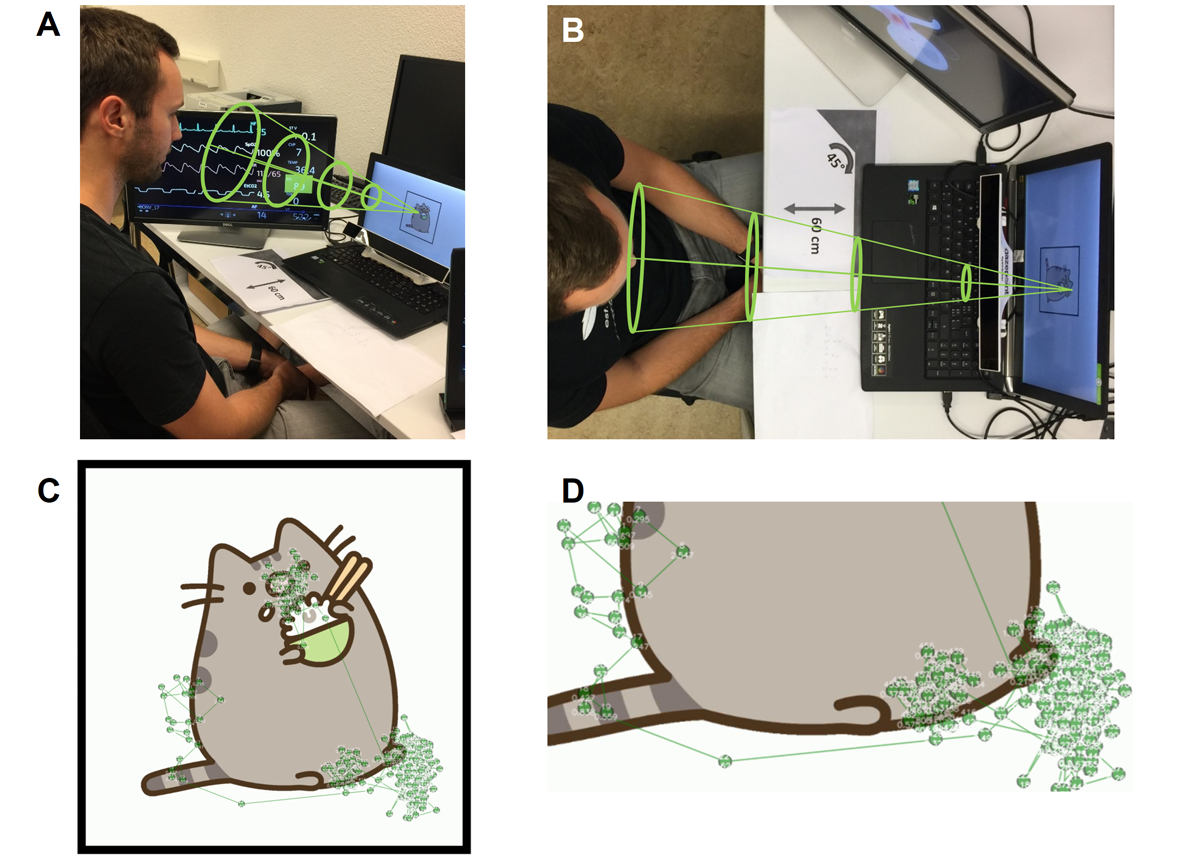
(A and B) Study setup: A study participant sits in front of 2 computer monitors. An eye tracker records the participant’s eye movements, which we used to confirm that the monitor on which the changes in patient condition were displayed was located in the peripheral field of view of the participant. The green funnel shows where the participant is looking and confirms that the monitor to the left remains in the peripheral visual field of the participant as long as they do not look away from the laptop screen in front. The base of the green cone corresponds to a radius of approximately 30° around the participant’s point of sharpest vision. Everything outside the funnel lies in the participant’s peripheral field of view. (C and D) The gaze plot data for 1 participant. Each point indicates a gaze fixation. A line links successive fixations.

Figure 2 shows a picture of the experimental setup. A video showing a complete peripheral vision scenario is available (Multimedia Appendix 1). We recorded this video with a wide-angle camera, which we placed in such a way that it is possible for the reader to repeat the experiment by just looking at the cat and trying to identify the patient status changes on the screen to the left. The test works most realistically on a large screen and corresponds to our setup when the reader scales the video so that the central laptop screen has a diagonal of 15 inches.

### Scenarios

This experiment aimed to find out how many changes in patient status the participants could detect by peripheral vision with the 2 technologies (ie, avatar-based and conventional patient monitoring). For the 11 most important vital signs in today’s clinical routine presented in our scenarios, there were 18 possible changes. Among them, 7 vital signs could become too high or too low (eg, blood pressure and pulse) and 4 vital signs could become abnormal only in 1 direction (eg, oxygen saturation). For the 2 technologies, we presented 36 scenarios in all. In these scenarios, all vitals remained normal for 5 seconds, after which one vital sign changed into the abnormal range. In the conventional scenarios, the changing vital signs were highlighted in yellow and an alarm tone sounded to allow for a high degree of realism. After each change, the data collector asked the test persons whether they had recognized which vital sign had just changed and, if yes, in which direction. The participants also indicated how confident they were that their assessment was correct. Participants were to choose from 0=very unconfident, 1=unconfident, 2=confident, and 3=very confident. In the videos, we showed the vital sign changes in randomized order, alternating avatar-based with conventional monitoring. To reduce the influence of the order in which the videos showed the vital sign changes, half of the participants evaluated a video in which we completely reversed the order of the vital sign changes compared with the first video.

### Outcome Measures

The primary objective of this study was to compare the performance of avatar-based monitoring with that of conventional patient monitoring in terms of the perception of patient status changes with peripheral vision. To quantify performance, we compared the number of recognized changes in vital signs with the 2 technologies. The higher the number of recognized vital sign changes, the more efficient the technology.

Secondary goals were to find out which vital signs the participants detected with the respective technologies and how confident they felt about the diagnoses they made.

### Statistical Analysis

As each participant evaluated the same vital sign changes using the 2 technologies, we used paired Student *t* test to check the differences for statistical significance. To compare subgroup data, we used Mann-Whitney U test, and for contingency tables, we used Fisher exact test, as appropriate.

### Sample Size

The sample size planning was based on the results of a pilot study and a post hoc sample size calculation for a paired *t* test. On the basis of these results, a sample size of 8 participants could demonstrate a difference in one of the 11 vital signs with a power of 80% at a significance level of 5%. In this calculation, we assumed that an improvement of a single perceived patient status change corresponds to the minimum clinically relevant difference.

For both scenarios, we expected significantly more than 8 participants and a higher difference than 1 patient status change between the technologies. Therefore, it was clear that with a total of 30 participants, the minimum requirements for a power of 80% at a significance level of 5% were exceeded.

## Results

### Study and Participant Characteristics

Table 1 shows the characteristics of this study and its participants in detail. Overall, 38 participants took part in the 2 study centers. Eye-tracking data were missing in 5 of the 38 participants because of technical recording problems. We excluded 3 more participants from the analysis because they looked to the left of the central monitor several times. In the end, we included data from 30 participants for evaluation. The 2 groups of participants at the Cantonal Hospital Winterthur and University Hospital Zurich were not significantly different in terms of gender, composition (professional groups), and anesthesia experience. The only difference was that participants from the Cantonal Hospital Winterthur more frequently belonged to a higher age group than did those from the University Hospital Zurich.

**Table 1:** Study and participant characteristics.

Name of study center with number of participants.		Cantonal Hospital Winterthur (n=22)	University Hospital Zurich (n=16)	*P* value
Participants included in data analysis, n (%)		17 (77)	13 (81)	>.99^a^
Senior anesthesiologists, n (%)		4 (25)	5 (38)	.69^a^
Resident physicians, n (%)		4 (25)	2 (15)	.66^a^
Nurse anesthetists, n (%)		8 (50)	6 (46)	>.99^a^
**Number of female/male participants, n (%)**				
	Female	9 (56)	8 (62)	.69^a^
	Male	7 (44)	5 (38)	.69^a^
Age group of participants (years), median (IQR^b^)		45-54 (25-34 to 45-54)	25-34 (25-34 to 35-44)	*.* 05^c^
Anesthesia experience group of participants (years), median (IQR)		More than 10 (5-10 to >10)	5 to 10 (1-5 to >10)	.32^c^
Duration of data collection (minutes), median (IQR)		77 (70-86)	76 (70-80)	.39^c^
Duration of peripheral vision experiment (minutes), median (IQR)		13.5 (12-15)	13 (12-15)	.43^c^

^a^Fisher exact test.

^b^IQR: interquartile range.

^c^Mann-Whitney U test.

### Eye-Tracking Results

The eye-tracking data acquisition worked well. The success rate was 87% (33/38 participants). The most common reason for technical problems was thick eyeglass lenses, which did not allow for a successful calibration of the eye tracker. According to the study protocol, we excluded these data from the analysis. We provide the eye-tracking gaze plots of all individual participants (Multimedia Appendix 2).

### Primary Outcome

**Figure 3:**
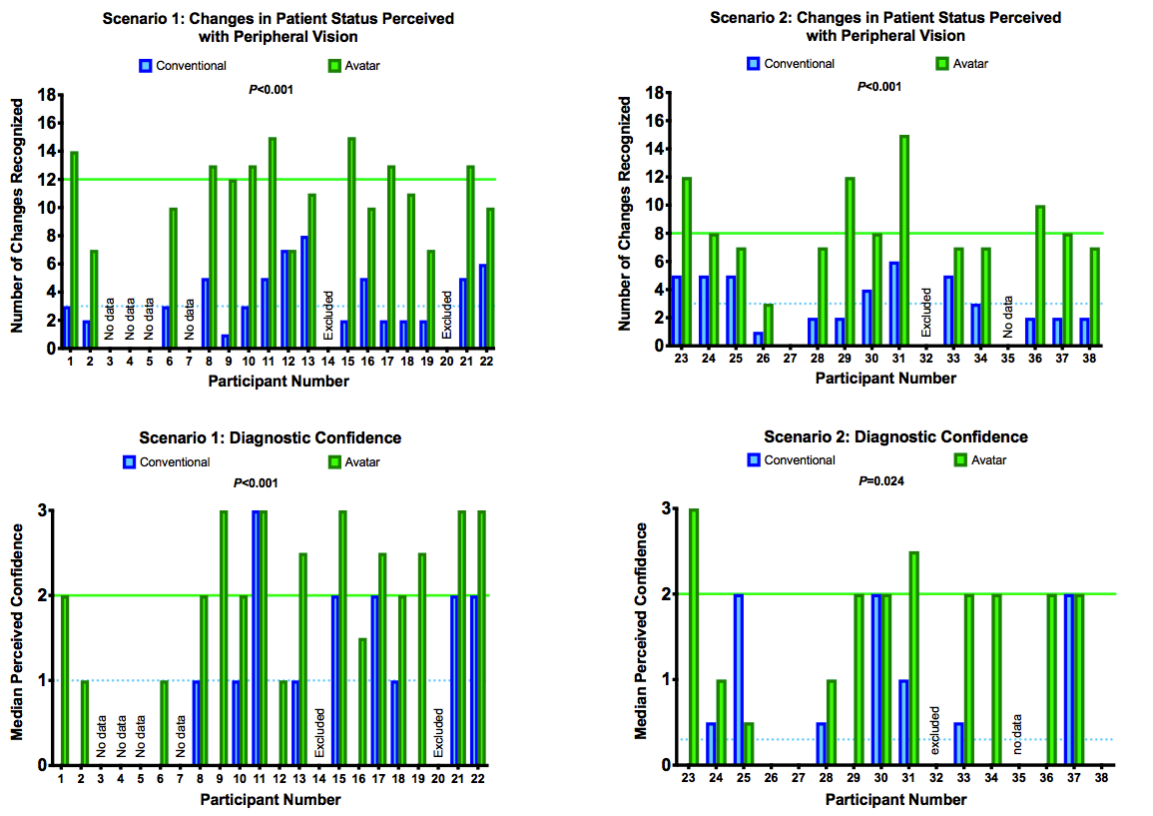
The results enabled 30 direct intraparticipant comparisons. All except 2 participants achieved a better performance with the avatar. The number of perceived changes in the patient’s condition quadrupled in scenario 1 and more than doubled in scenario 2. Median perceived confidence: 0=very unconfident, 1=unconfident, 2=confident, and 3=very confident. Paired Student t tests showed statistical significance for all results.

When the avatar was used, the number of changes in the patient’s condition noticed with peripheral vision was higher. In scenario 1, it was higher by 9 vital signs, rising from a median (with interquartile range) of 3 (2-5) with the conventional monitor to 12 (10-13) with the avatar-based monitor (*P*<.001). In scenario 2, it was higher by 5 vital signs: increasing from 3 (2-5) with the conventional monitor to 8 (7-11) with the avatar-based monitor (*P*<.001). Figure 3 shows these results on individual participant level.

Only 2 of 30 participants achieved the same result with conventional monitoring as with avatar-based patient monitoring. No participant performed better with conventional monitoring.

With conventional monitoring, only 2 changes in the patient’s condition could be detected by more than half of the participants: *pulse too high* and *pulse too low*.

With the avatar, more than half of the participants recognized the following 8 out of 18 vital sign changes: (1) *pulse rate too high*, (2) *blood pressure too high*, (3) *saturation too low*, (4) *central venous pressure too high*, (5) *expiratory carbon dioxide concentration too high*, (6) *respiratory rate too high*, (7*) body temperature too high*, and (8) *body temperature too low*.

Only the vital sign change *pulse too low* was recognized by more participants with conventional monitoring than with avatar-based monitoring (Fisher exact test *P*<.001).

Table 2 shows exactly how many participants recognized each patient status change with each technology.

**Table:** 

Vital sign	Scenario 1 (n=16)			Scenario 2 (n=13)		
	Conventional, n (%)	Avatar, n (%)	*P* value^a^	Conventional, n (%)	Avatar, n (%)	*P* value^a^
Pulse too high	16 (100)	16 (100)	>.99	13 (100)	12 (92)	>.99
Pulse too low	8 (50)	0 (0)	.002	9 (69)	1 (8)	<.004
Blood pressure too high	0 (0)	16 (100)	<.001	1 (8)	13 (100)	<.001
Blood pressure too low	4 (25)	4 (25)	>.99	0 (0)	6 (46)	.010
Saturation too low	10 (63)	15 (94)	.080	2 (15)	9 (69)	.020
Central venous pressure too high	2 (13)	15 (94)	<.001	0 (0)	7 (54)	.005
Central venous pressure too low	0 (0)	10 (63)	<.001	1 (8)	2 (15)	>.99
ST-Segment abnormal	4 (25)	7 (44)	.46	4 (31)	2 (21)	.64
Expiratory carbon dioxide concentration too high	4 (25)	16 (100)	<.001	3 (21)	13 (100)	<.001
Expiratory carbon dioxide concentration too low	5 (31)	10 (63)	.16	2 (15)	1 (8)	>.99
Respiratory rate too high	1 (6)	14 (88)	<.001	3 (23)	11 (85)	.005
Respiratory rate too low	2 (13)	2 (13)	>.99	2 (15)	2 (15)	>.99
Tidal volume too high	2 (13)	15 (94)	<.001	1 (8)	6 (46)	.070
Tidal volume too low	2 (13)	2 (13)	>.99	1 (8)	1 (8)	>.99
Brain activity high	2 (13)	9 (56)	.020	0 (0)	5 (38)	.040
Body temperature too high	2 (13)	16 (100)	<.001	0 (0)	12 (92)	<.001
Body temperature too low	0 (0)	9 (56)	<.001	0 (0)	7 (54)	.005
Neuromuscular relaxation high	0 (0)	5 (31)	.040	0 (0)	2 (15)	.48

^a^Fisher exact test.

### Secondary Outcomes

The participants’ perceived confidence in the correctness of their diagnoses reflected the higher number of perceived changes in the patient’s condition. Only one of the 30 participants rated perceived confidence higher with conventional monitoring than with avatar-based monitoring. In scenario 1, median perceived confidence in the correctness of the diagnoses was *1*, that is, *unconfident* with conventional monitoring and *2*, that is, *confident* with avatar-based monitoring (*P*<.001). In scenario 2, this was *3*, that is, *very unconfident* with conventional monitoring and *2*, that is, *confident* with avatar-based monitoring (*P*<.001).

## Discussion

### Principal Findings

In this study, we found substantial differences between avatar-based and conventional patient monitoring with peripheral vision. In avatar-based monitoring, more than half of the participants in both scenarios detected the following 8 changes in patient status: (1) *pulse rate too high*, (2) *blood pressure too high*, (3) *oxygen saturation too low*, (4) *central venous pressure too high*, (5) *expiratory carbon dioxide concentration too high*, (6) *respiratory rate too high*, (7) *body temperature too high*, and (8) *body temperature too low*. In conventional patient monitoring, the only 2 changes that more than half of the participants in both scenarios detected were (1) *pulse rate too high* and (2) *pulse rate too low*. The pulse rate *too low* signal was the only one of the 18 total vital sign changes that was better detected with conventional monitoring (Table 2). Anesthesia providers are trained to detect a *too slow* pulse rate via the acoustic pulse tone because it implies serious a problem in a real patient. However, a nonpulsating avatar, as is the case for a low pulse rate, was not as well detected with peripheral vision. This finding suggests that optimal information transmission could be achieved with a combination of auditory and avatar-based monitoring; it also emphasized the potential benefits of further development of audio displays in patient monitoring, as also found previously [31].

### Potential Significance of Peripheral Vision for Patient Monitoring

Ford et al showed that anesthetists only looked directly at the screen of the patient monitor for about 5% of the time during anesthesia cases. This means that anesthesia providers spend much of their time at an angle where patient monitoring with peripheral vision would in theory be possible [18,19].

On the one hand, monitoring with peripheral vision has the theoretical advantage that a care provider can determine which vital signs lie outside the patient’s normal range without having to look away from the patient or current tasks. This easing of workload would already be an advantage over today’s industry standard monitoring devices and would become even more of an advantage with an augmented-reality head-mounted monitoring device in the future [32,33]. On the other hand, even during foveal viewing of the avatar, the information around the point of sharpest vision can still be perceived in parallel using peripheral vision. This method of information reception is not possible when reading a number from a conventional patient monitor interface, as humans can only read and process individual numbers in sequence [10,25]. Various sources have suggested that it is desirable for patient safety and operator well-being to design the exchange of information between an instrument and its human user as efficiently as possible [9,18,22]. A caregiver can only make the right decision for a patient if situation awareness is high. By definition, the concept of situation awareness encompasses 3 levels: (1) perception of elements in the environment within a volume of time and space, (2) understanding their meaning, and (3) projection of their status into the near future [13,22,34,35]. A lack of situational awareness prohibits sound decision making and is increasingly recognized as the cause of incidents and accidents in the medical field and in aviation [13-15,22,36]. Inadequate situation awareness constitutes a hole in the Swiss cheese model of Reason’s theory of human error causation [37]. For patients who are connected to a patient monitor, the real-time and trend information from the screens and the acoustic displays are essential to the caregiver’s situation awareness. Alarm fatigue and too frequent alarms are a major problem for anesthesia providers in their daily interaction with patient monitoring [17,38-40]. Avatar-based monitoring could theoretically provide a way to reduce audible alarms if, for example, an initial alarm was only visual and would only trigger an audible alarm after some time without a reaction.

The high ratings of the subjectively perceived certainty that their diagnoses were correct shows that the users had confidence in their assessment, despite the fact that they had only seen it with their peripheral vision and therefore hazily.

This study showed us that there are numerous potential advantages of patient monitoring by means of peripheral vision and that an animated patient avatar appears to be a good tool for evaluating the real-life usability of the concept of monitoring with peripheral vision. We plan to conduct further studies along this line.

### Limitations

We conducted this study as a computer-based laboratory study; therefore, it has limitations.

For instance, we have not yet tested the avatar in a real operating room and have not evaluated any clinical patient outcomes, such as clinical status of patients after surgery or adverse events. Although only a study conducted in a real-life environment or a high-fidelity simulator will ultimately allow for confident conclusions about the true benefits of avatar-based and peripheral vision monitoring, it is plausible that the large and reliable intraindividual improvements we observed would also manifest outside of the laboratory. In addition, we are at a very early stage of concept development, where it is crucial to identify potential theoretical benefits to determine whether clinical use is ultimately warranted.

In this study, we used a realistic simulation of a conventional monitor for conventional patient monitoring, including audio alarms and color highlighting of pathological values, such as the state-of-the-art devices that are currently in routine clinical use. It would be theoretically conceivable that further developments of these devices, which make pathological vital signs larger than *normal* vitals, would also be better readable with peripheral vision than today’s devices. Nevertheless, even with critical numbers larger than the other numbers on a monitor, an avatar might have advantages because theoretically, although it is not yet tested, several vital signs could be readable simultaneously.

Another limitation is that we did not randomize the selection of participants for this study but that we recruited participants according to availability. However, we followed a plan made before the beginning of the study to include equal numbers of male and female participants and to balance participant numbers from the different occupational groups in the 2 centers. This standardization ensured that the groups were representative for all personnel groups and reduced the risk of sampling errors. Furthermore, the tendency of our anesthesia provider participants to look at the monitoring scenarios with foveal vision turned out to be low. Indeed, we only had to exclude 3 of 33 participants (10%) for looking at the peripheral monitor more than twice with foveal vision. All of the included participants looked at the peripheral monitor twice or less. Even if these 2 views of the monitor had allowed the perception of 2 status changes with foveal vision, this would have only affected 5% of the participants data, as each participant evaluated a total of 36 status changes. The low percentage of excluded participants and the eye-tracking method applied to confirm that the participants watched the scenarios with peripheral vision increase the validity of the study by reducing the risk of selection bias.

Finally, an inherent limitation of the avatar design that we would like to mention is the preprocessing of vital signs into categories, which causes a reduction of data accuracy. For example, pulse rate in the avatar can assume one of only 3 individual states, that is, *too low*, *normal*, or *too high*. Conversely, number and waveform monitoring can assume about 300 individual states between 0 and 300. Therefore, the avatar cannot replace routine monitoring, but it may serve as a supplement that explicitly aims at enhancing situation awareness.

### Conclusions

This study introduces the concept of peripheral vision monitoring. It provides empirical evidence that an avatar-based instrument can significantly improve the perception of patient status through peripheral vision. Further studies using the technology in real-life situations are necessary to show whether the benefits found can be translated into reality. This study represents a further building block in the literature on avatar-based monitoring by presenting and validating a hitherto unknown characteristic of the technology, namely, patient monitoring by means of peripheral vision.

## Appendix

Multimedia Appendix 1 A video showing the complete peripheral vision test.

Multimedia Appendix 2 The eye-tracking gaze plots of all individual study participants.
