# Supplementary material for: Avatar-Based Patient Monitoring With Peripheral Vision: A Multicenter Comparative Eye-Tracking Study
Source: J Med Internet Res. 2019 Jul 17;21(7):e13041. doi: 10.2196/13041 (PMC6668297; doi:10.2196/13041)

# Supplementary material

**Avatar-based patient monitoring with peripheral vision.**

# Gazeplots

# Scenario 1

# KSW Scenario 1 (Participant 1)

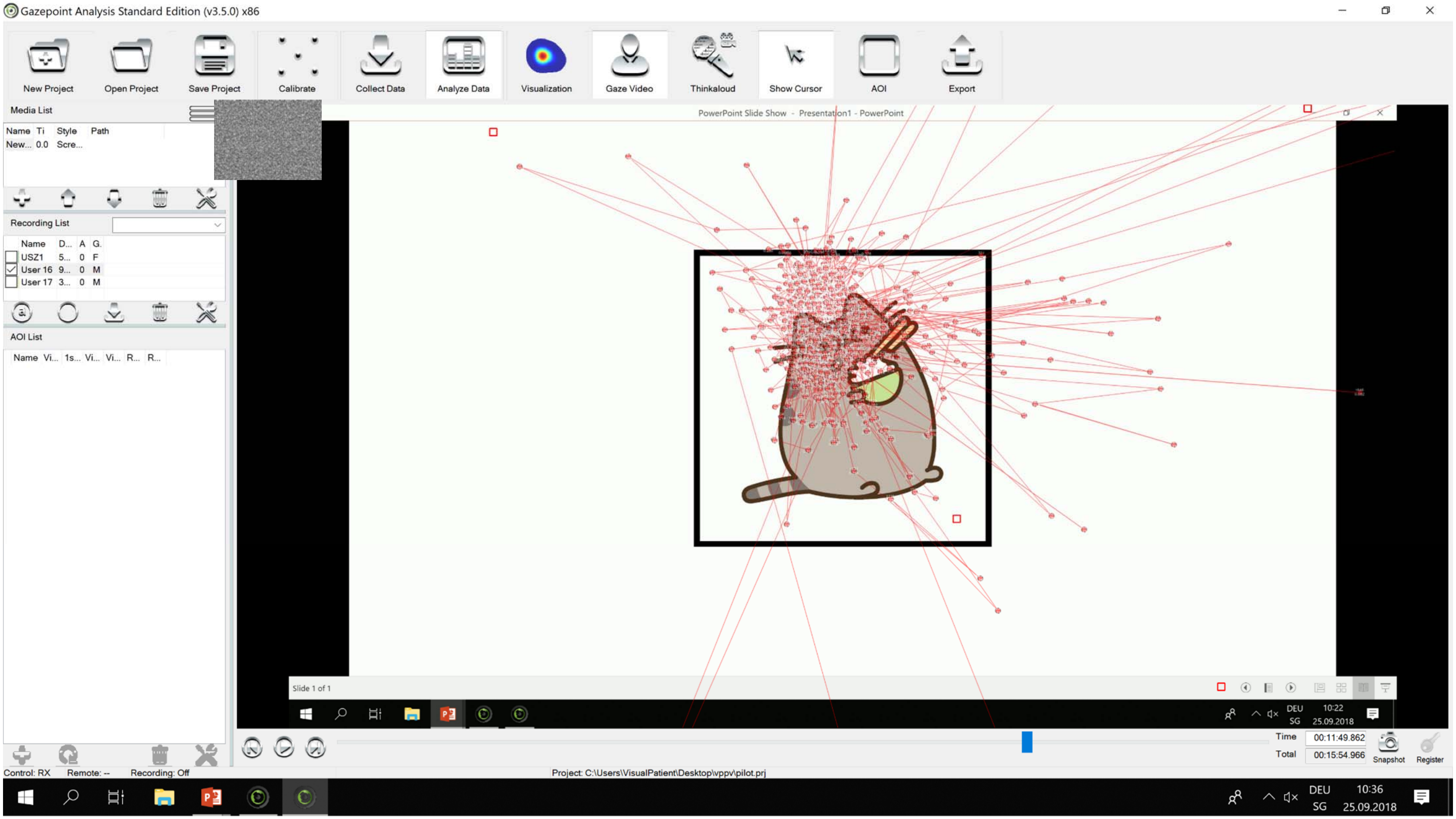

## KSW Scenario 1 (Participant 2)

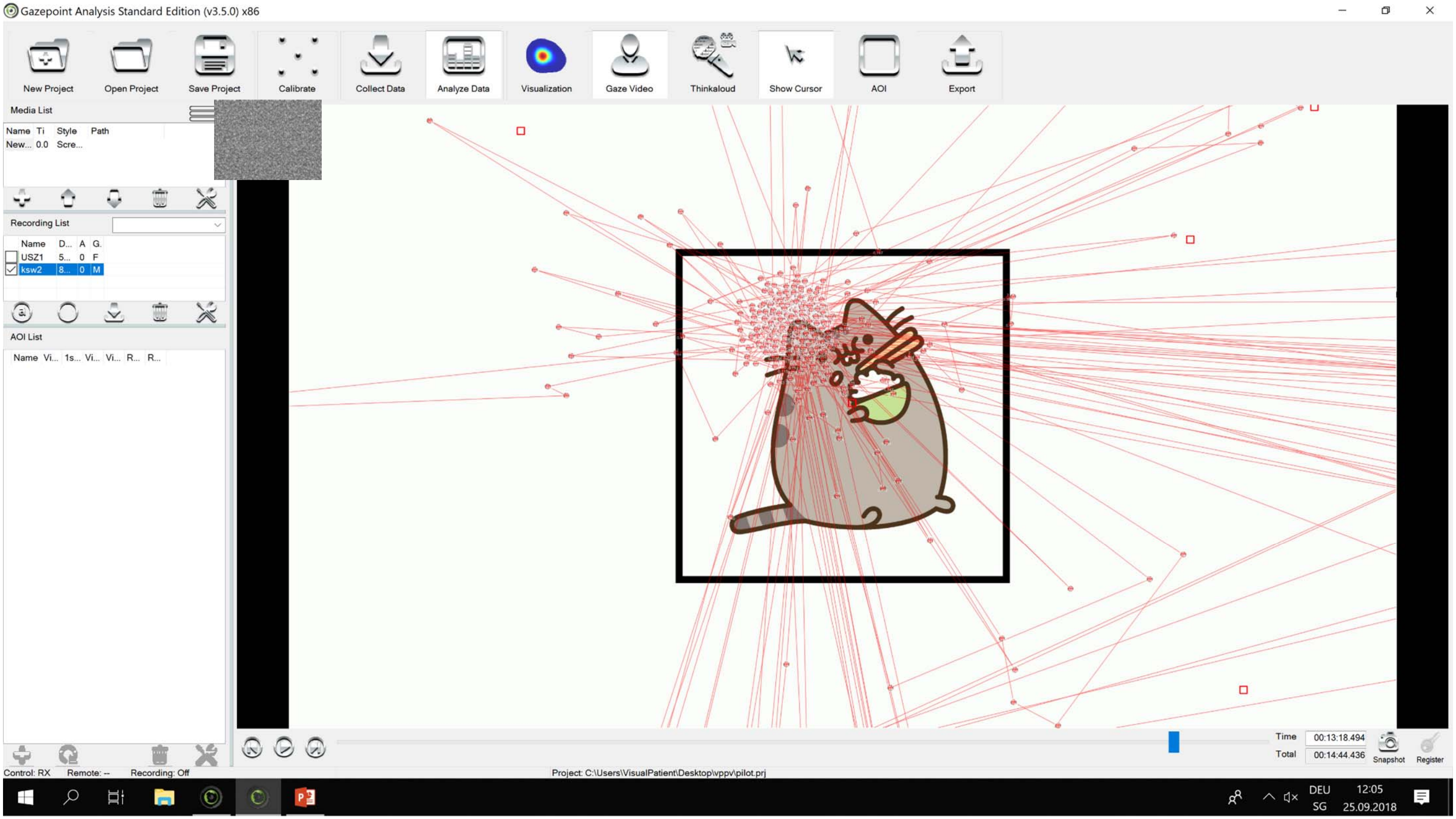

## KSW Scenario 1 (Participant 3)

- No data

## KSW Scenario 1 (Participant 4)

- No data

## KSW Scenario 1 (Participant 5)

- No data

# KSW Scenario 1 (Participant 6)

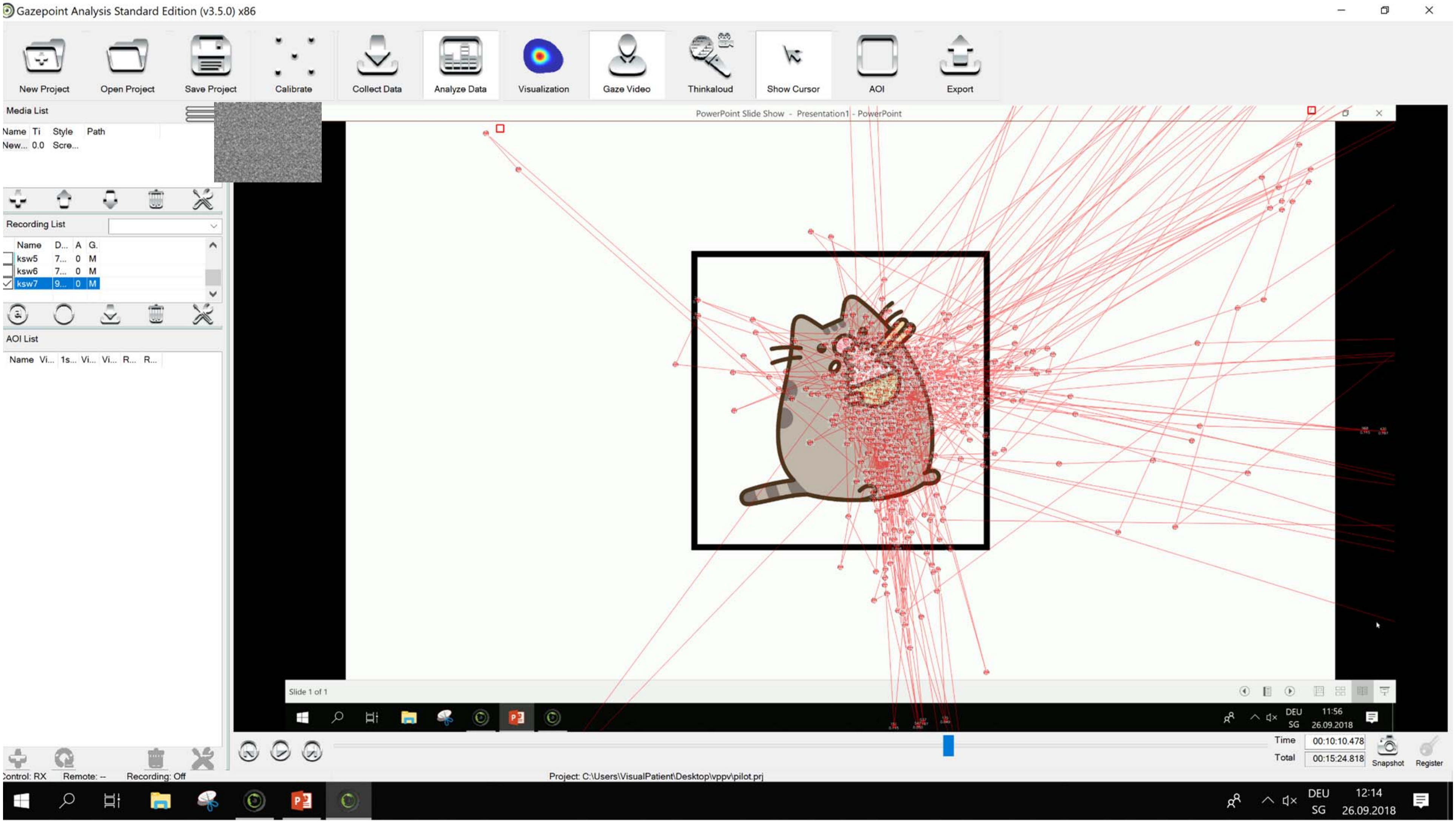

## KSW Scenario 1 (Participant 7)

- No data

# KSW Scenario 1 (Participant 8)

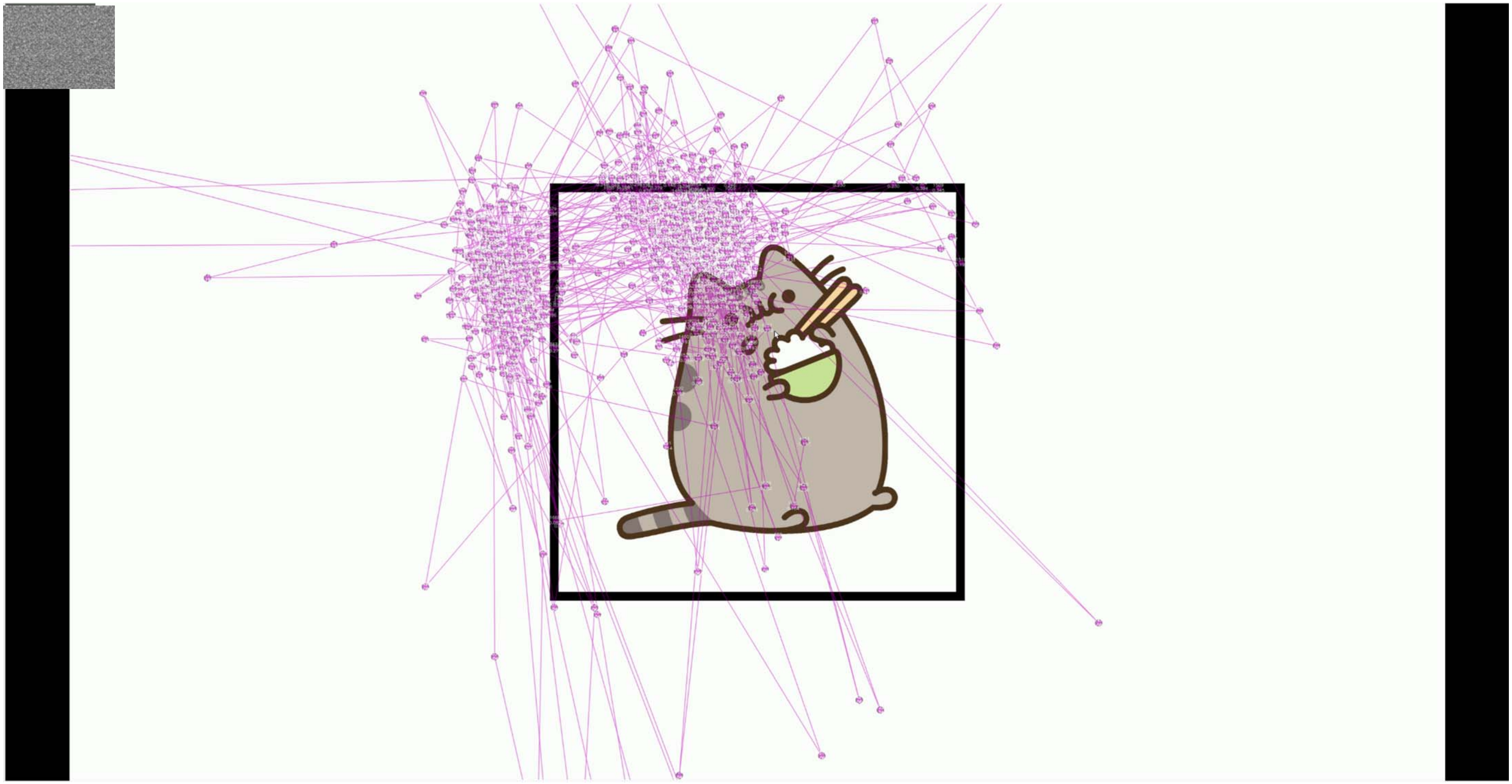

# KSW Scenario 1 (Participant 9)

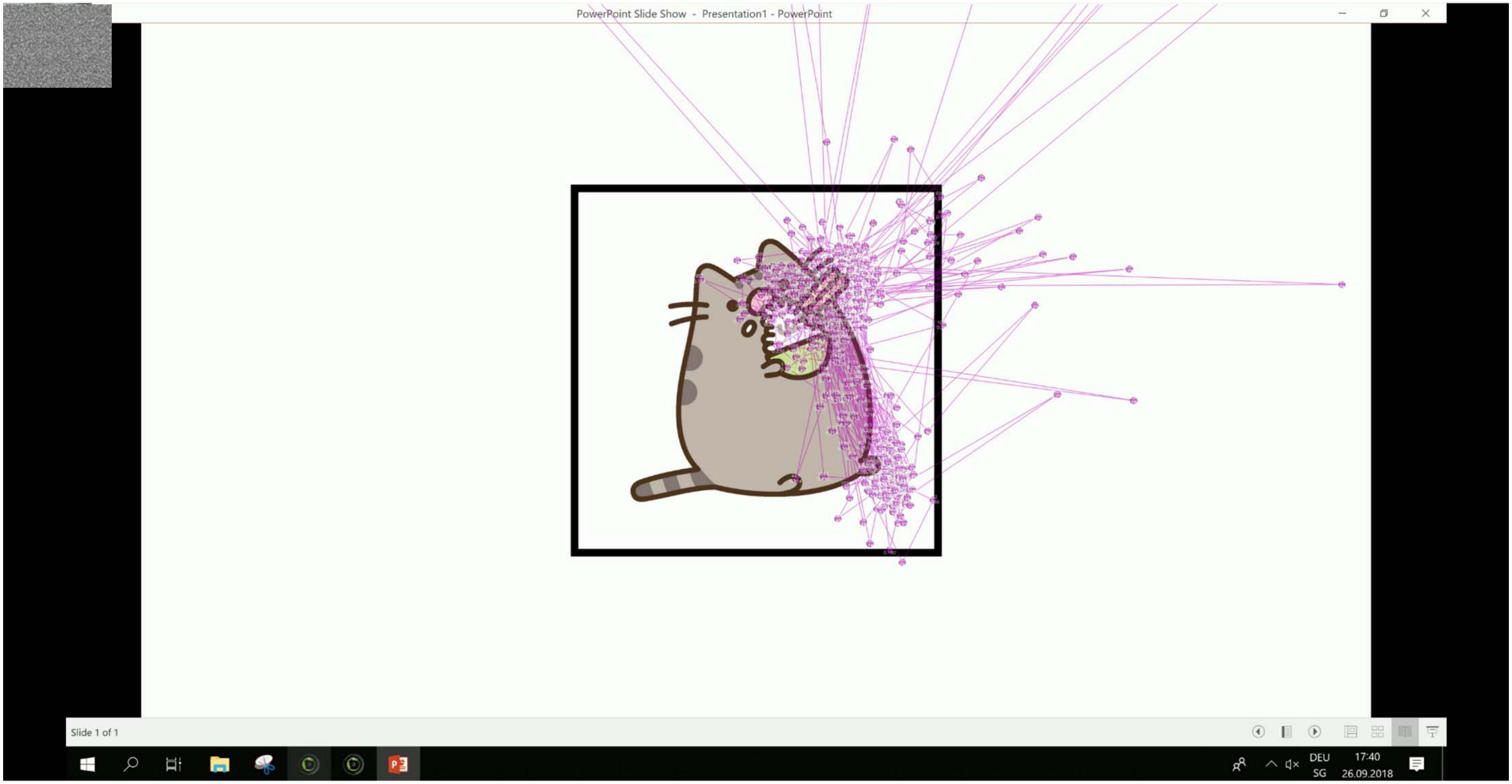

# KSW Scenario 1 (Participant 10)

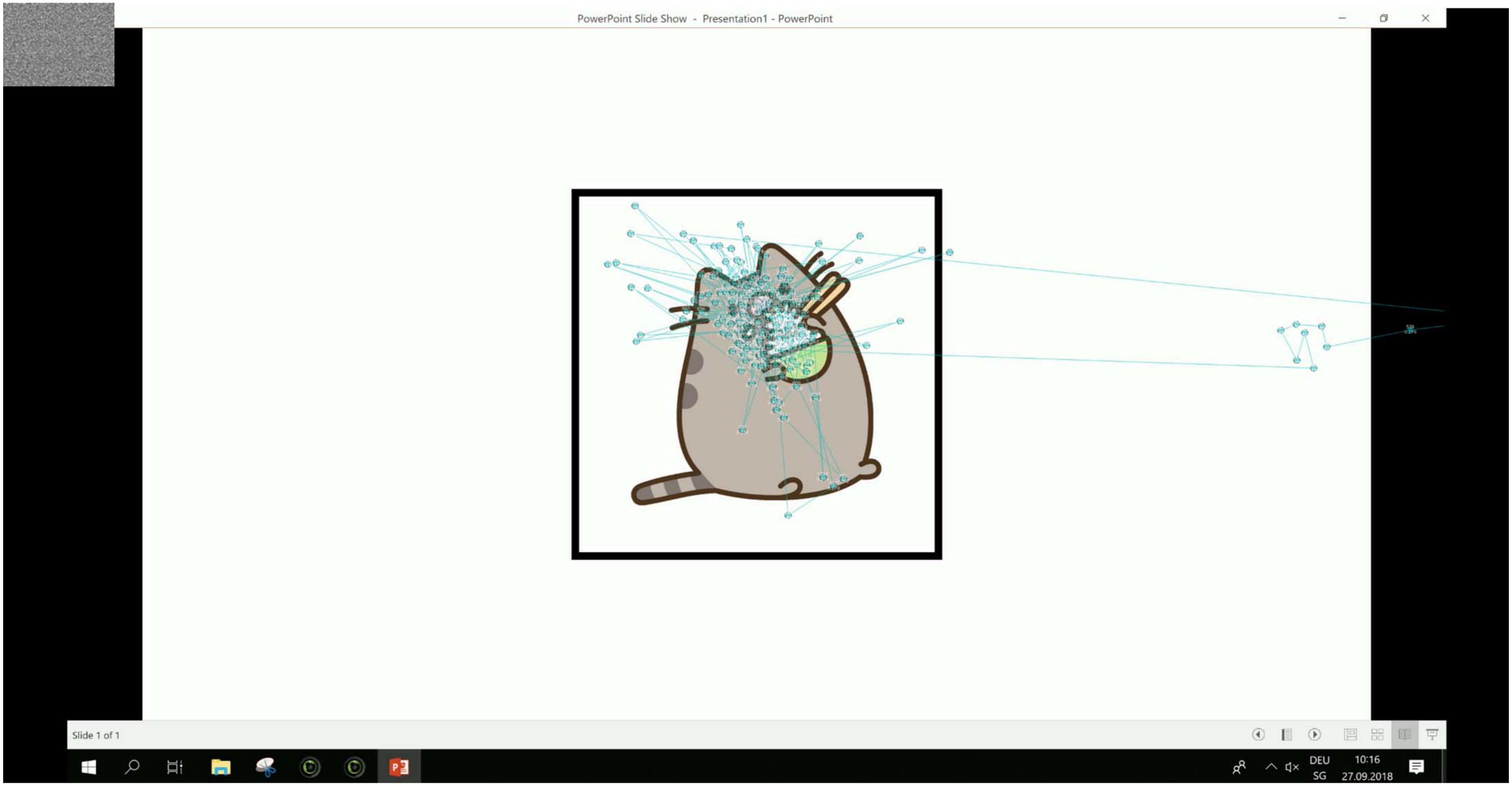

# KSW Scenario 1 (Participant 11)

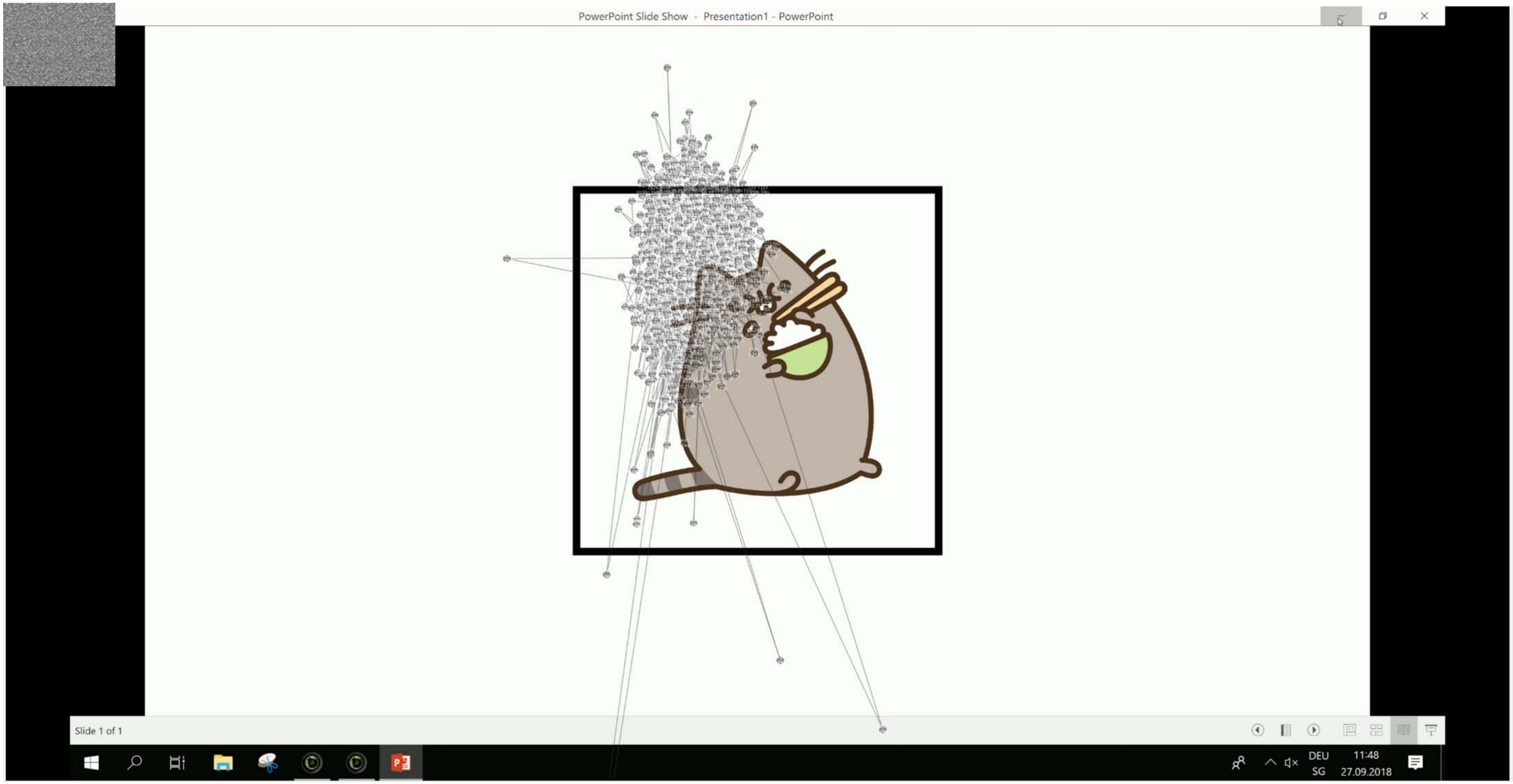

# KSW Scenario 1 (Participant 12)

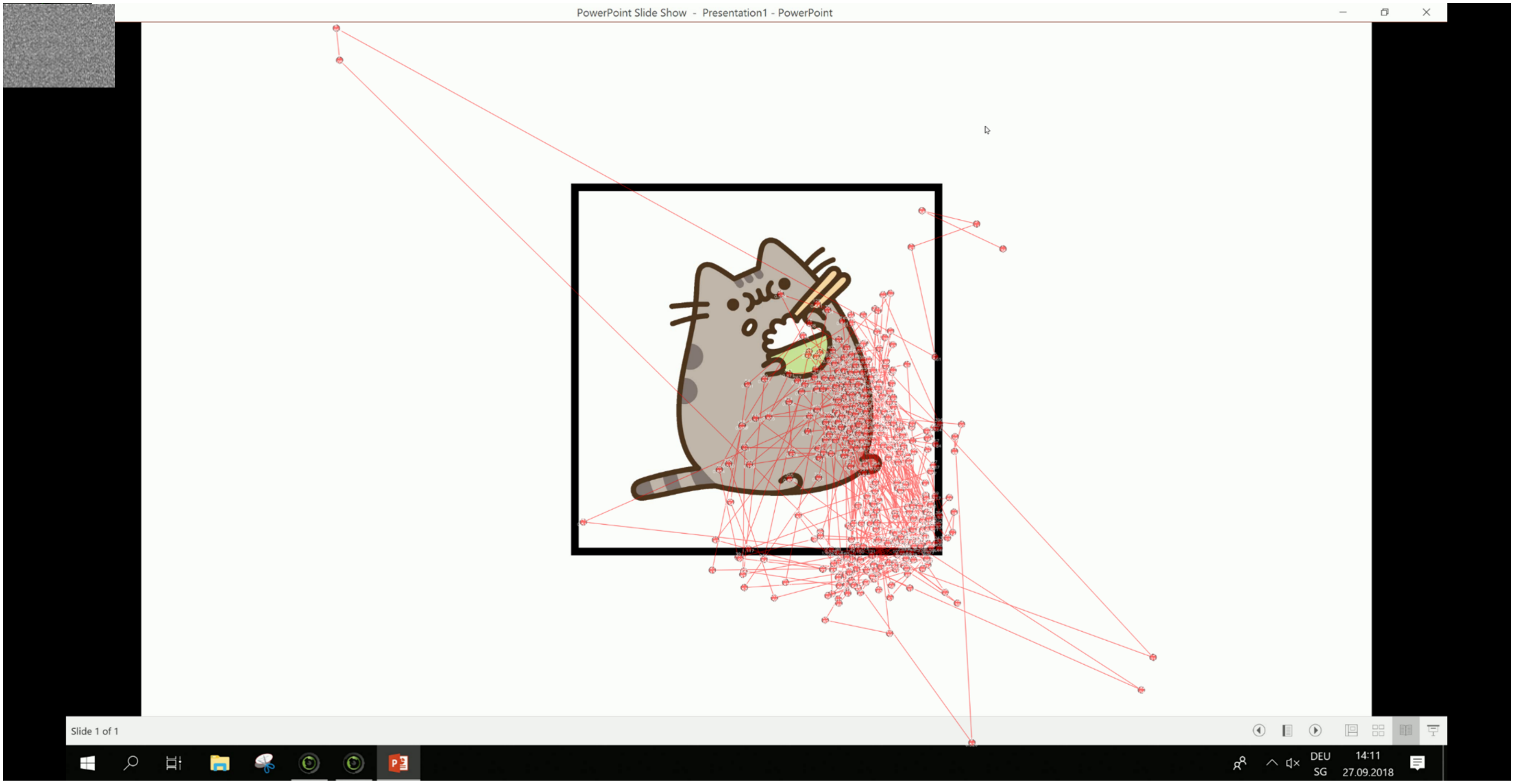

# KSW Scenario 1 (Participant 13)

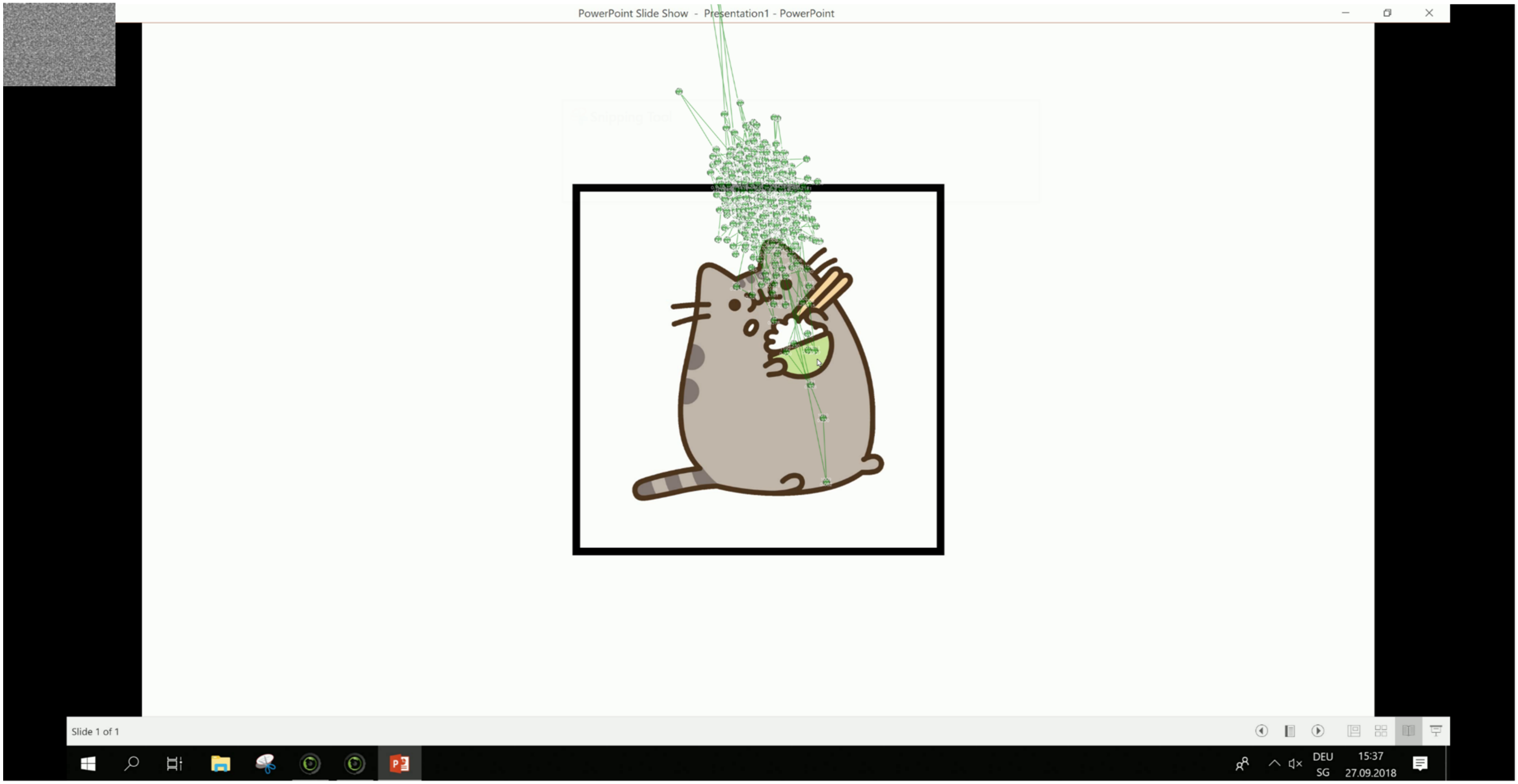

KSW Scenario 1 (Participant 14)

<u>Not included</u> in the data analysis due to view to the left.

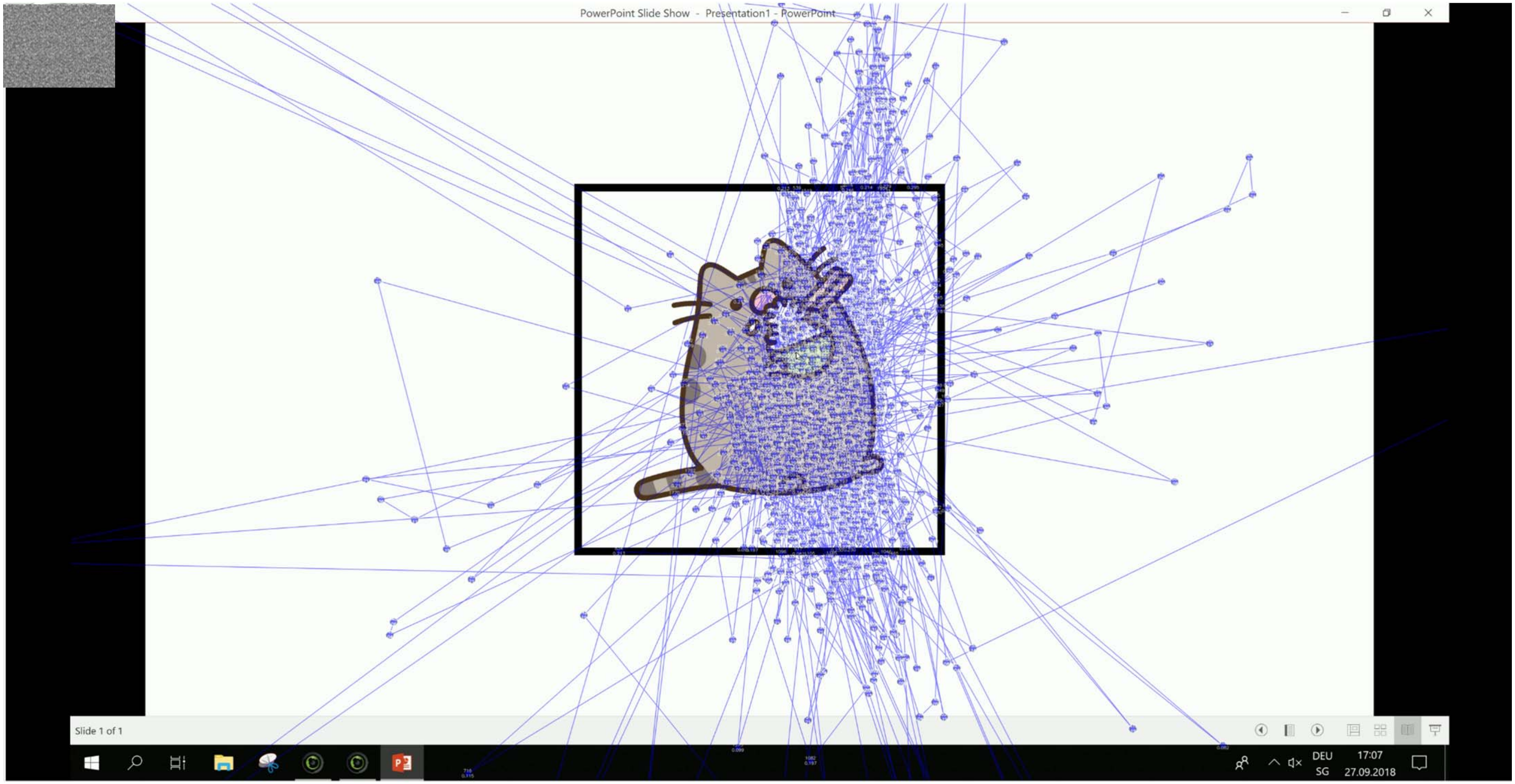

# USZ Szenario 1 (Participant 15)

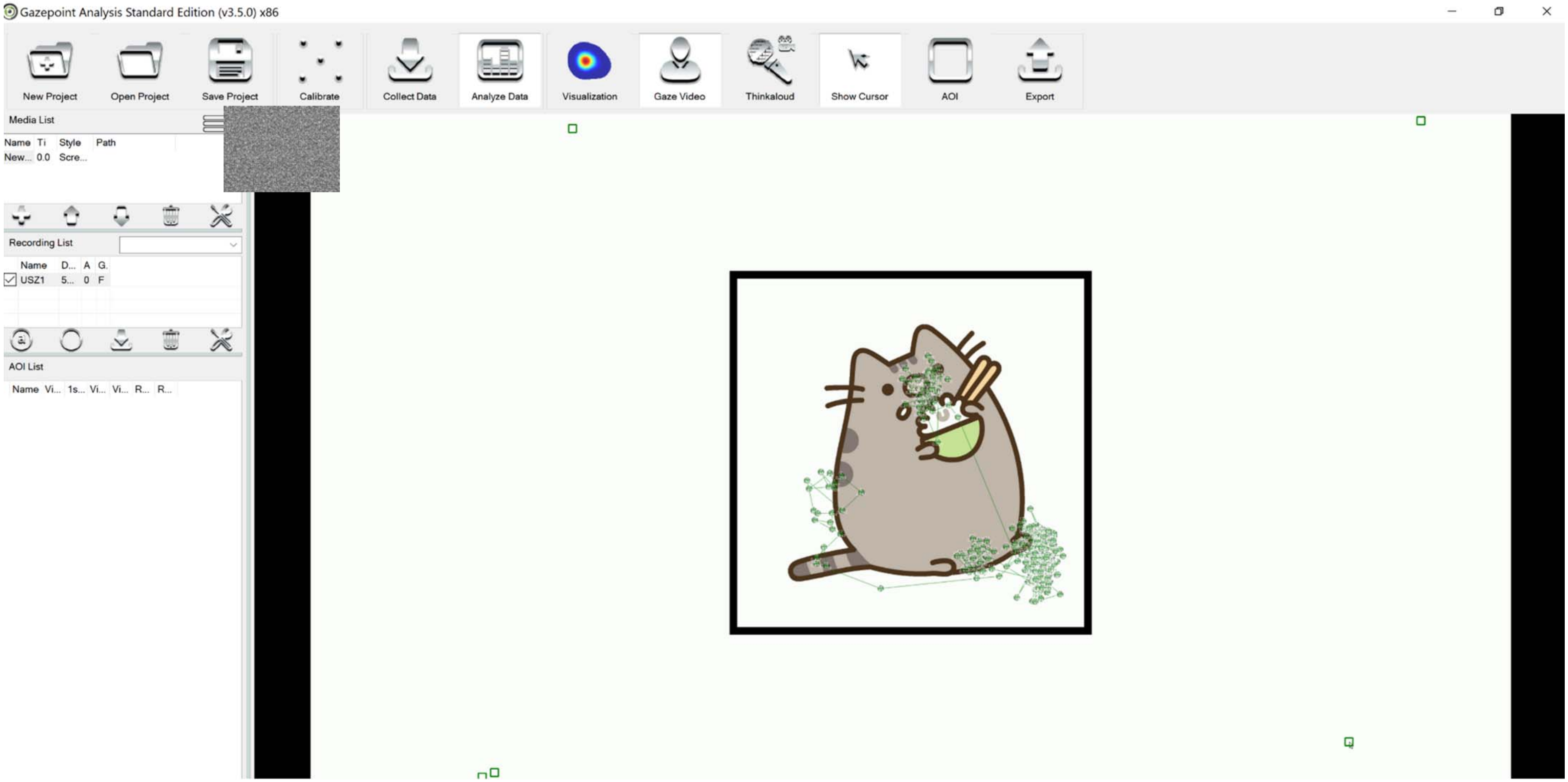

# USZ Szenario 1 (Participant 16)

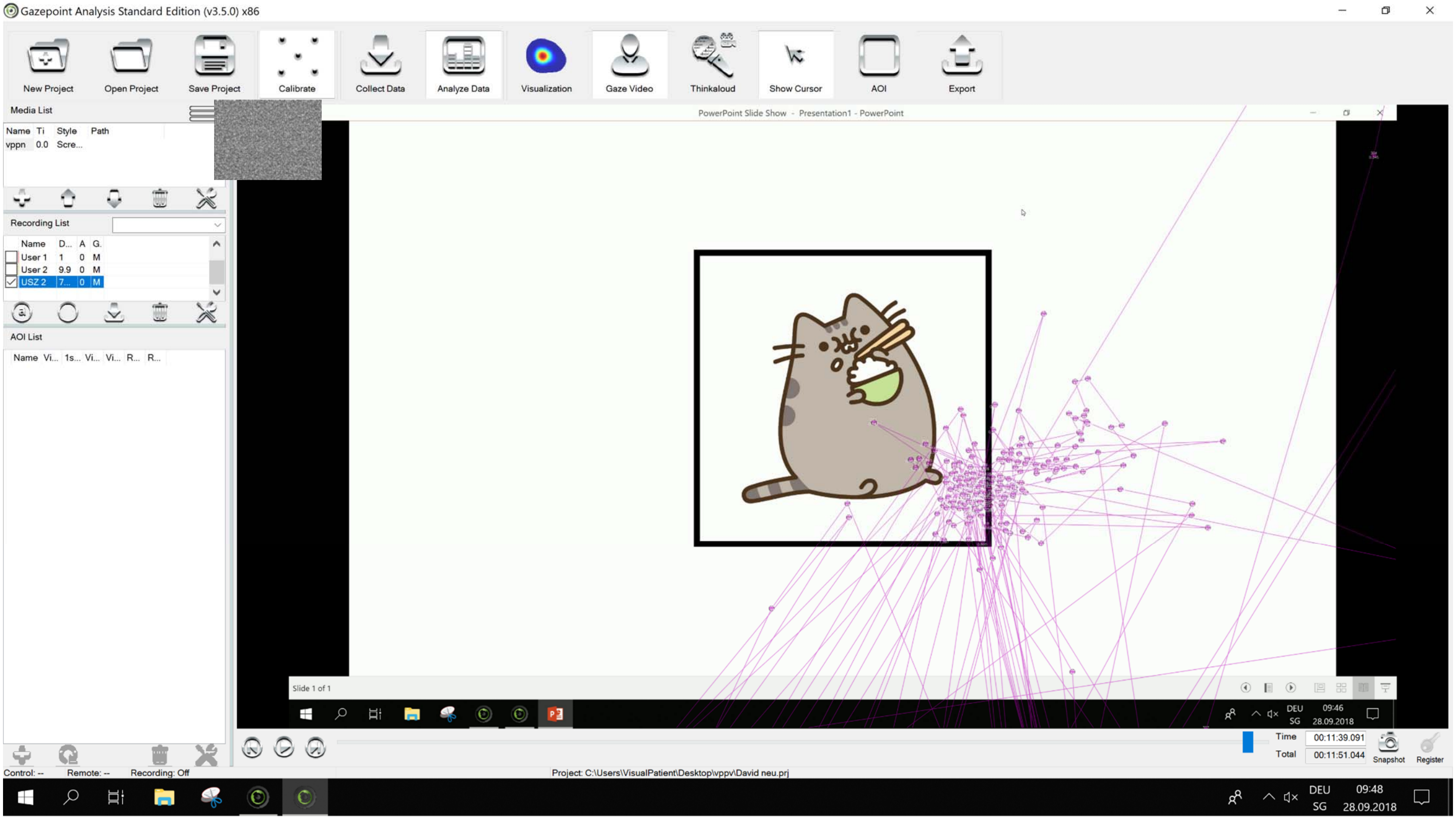

# USZ Szenario 1 (Participant 17)

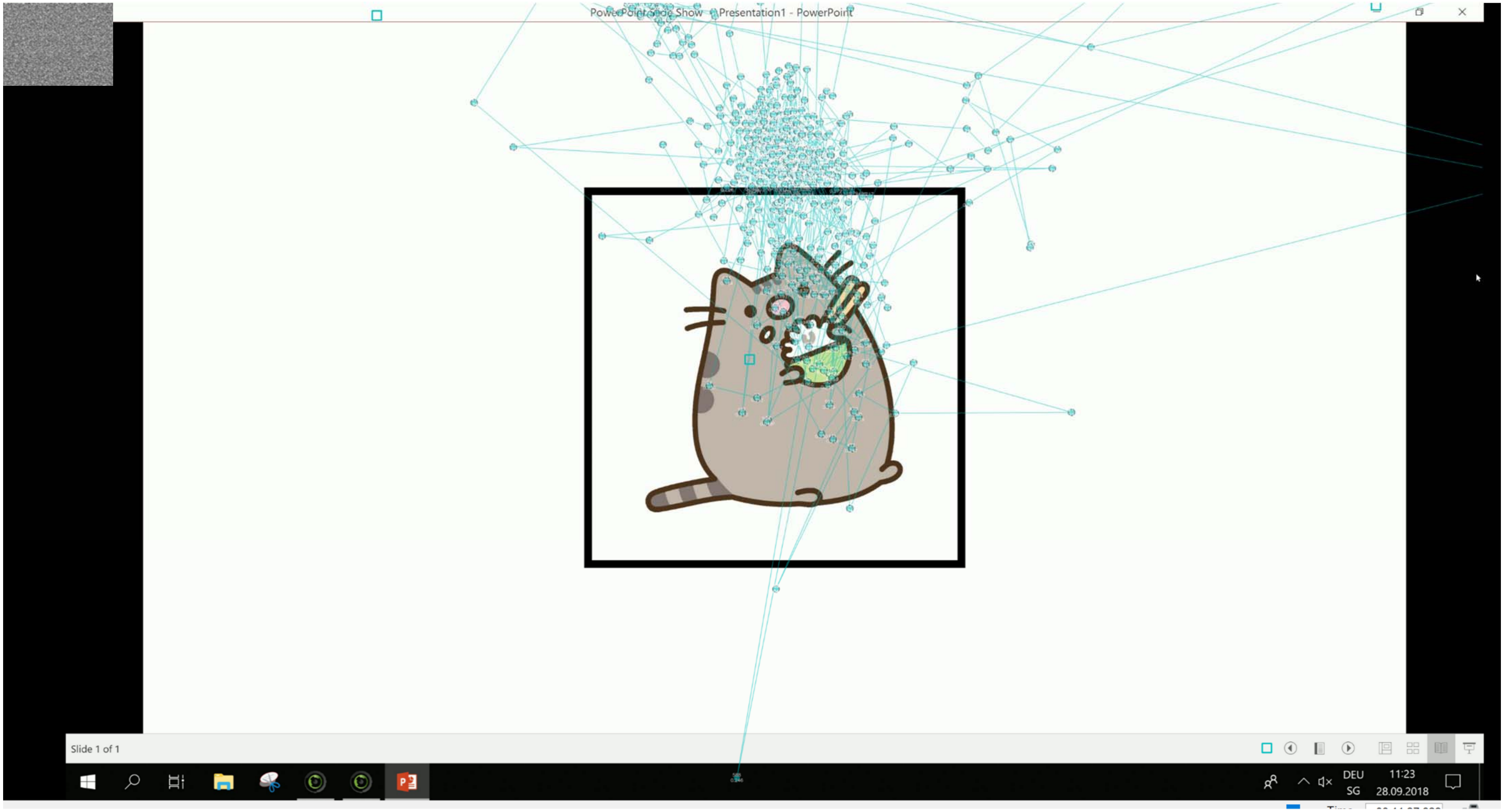

# USZ Szenario 1 (Participant 18)

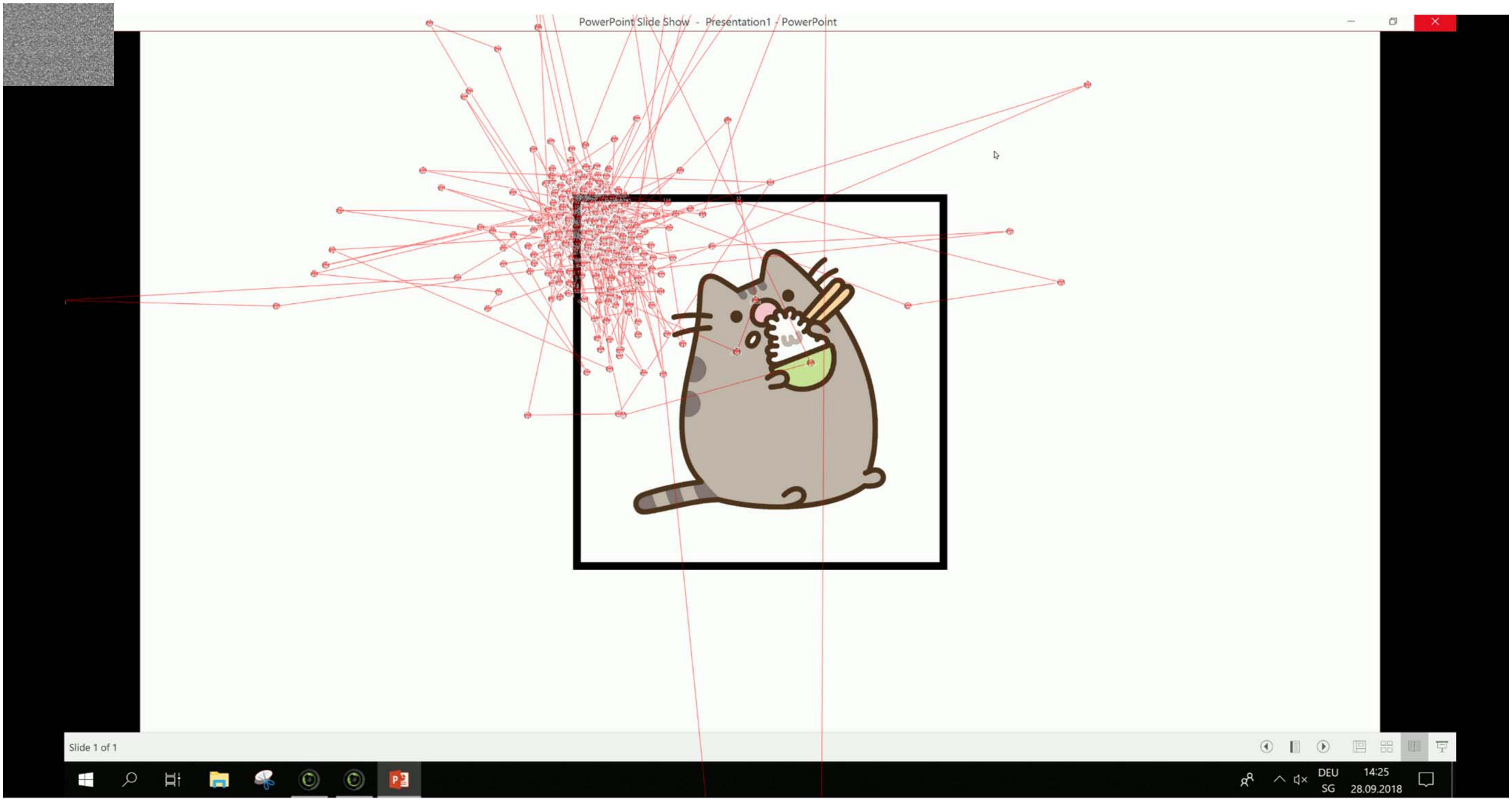

# USZ Szenario 1 (Participant 19)

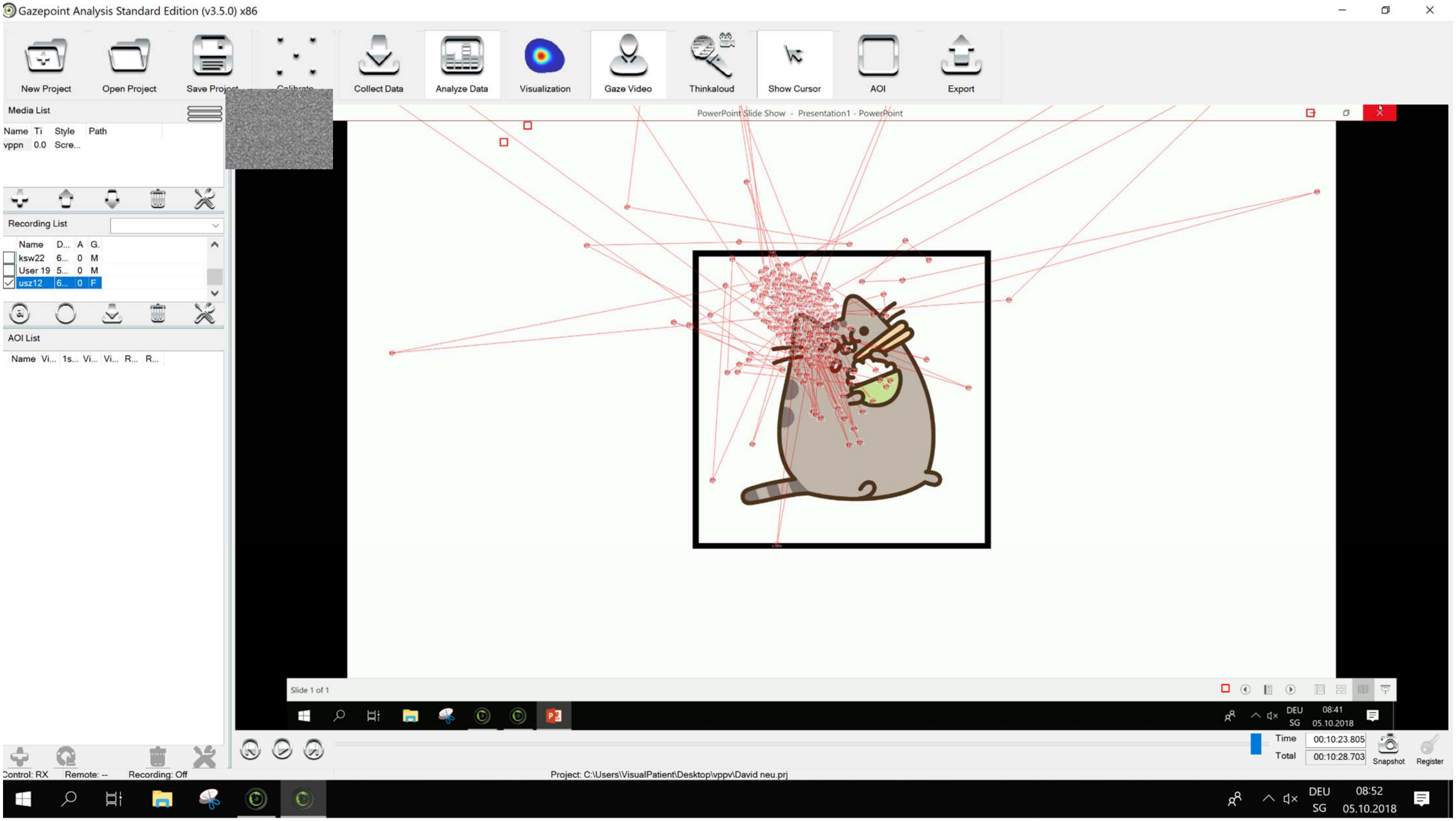

## USZ Scenario 1 (Participant 20)

Not included in the data analysis due to view to the left.

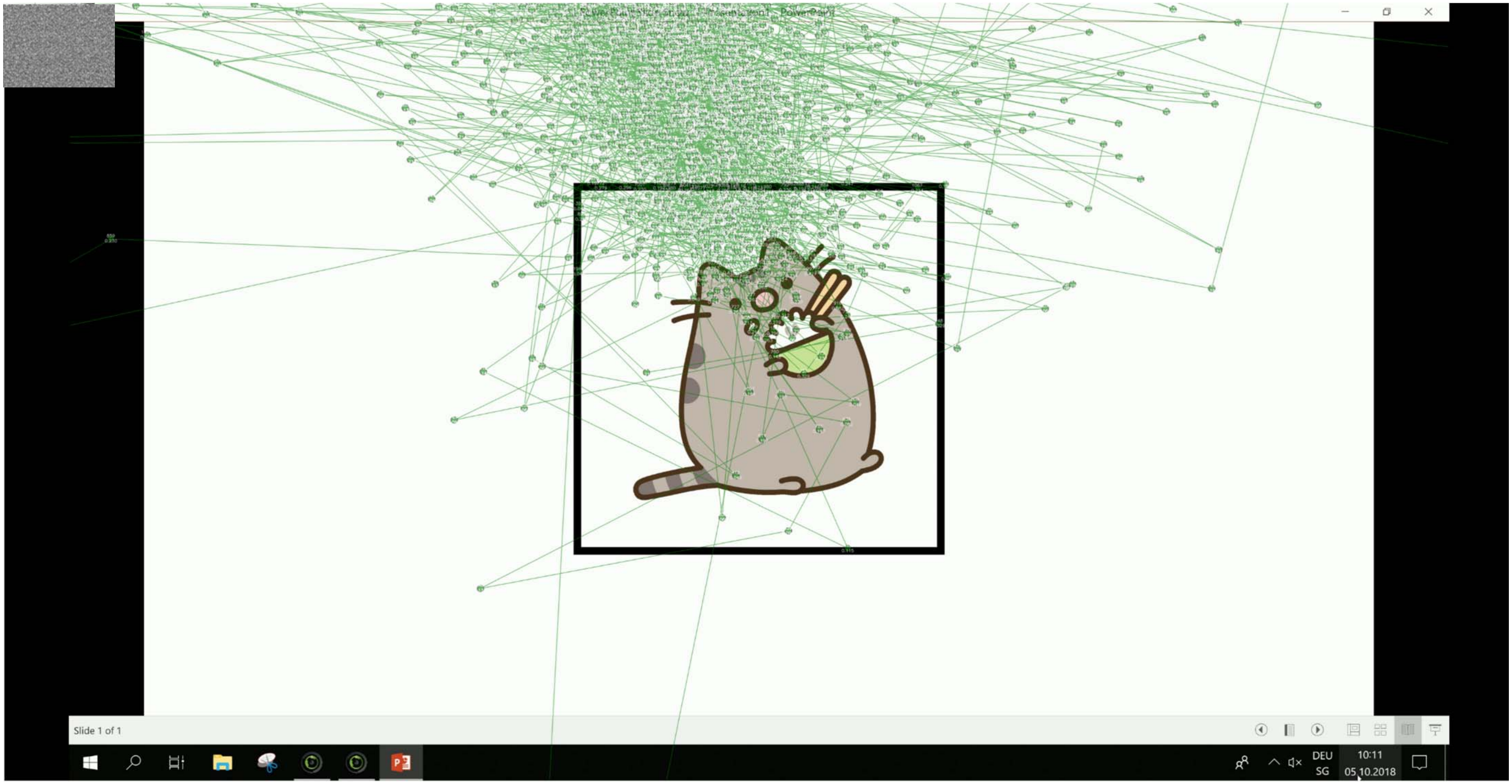

# USZ Szenario 1 (Participant 21)

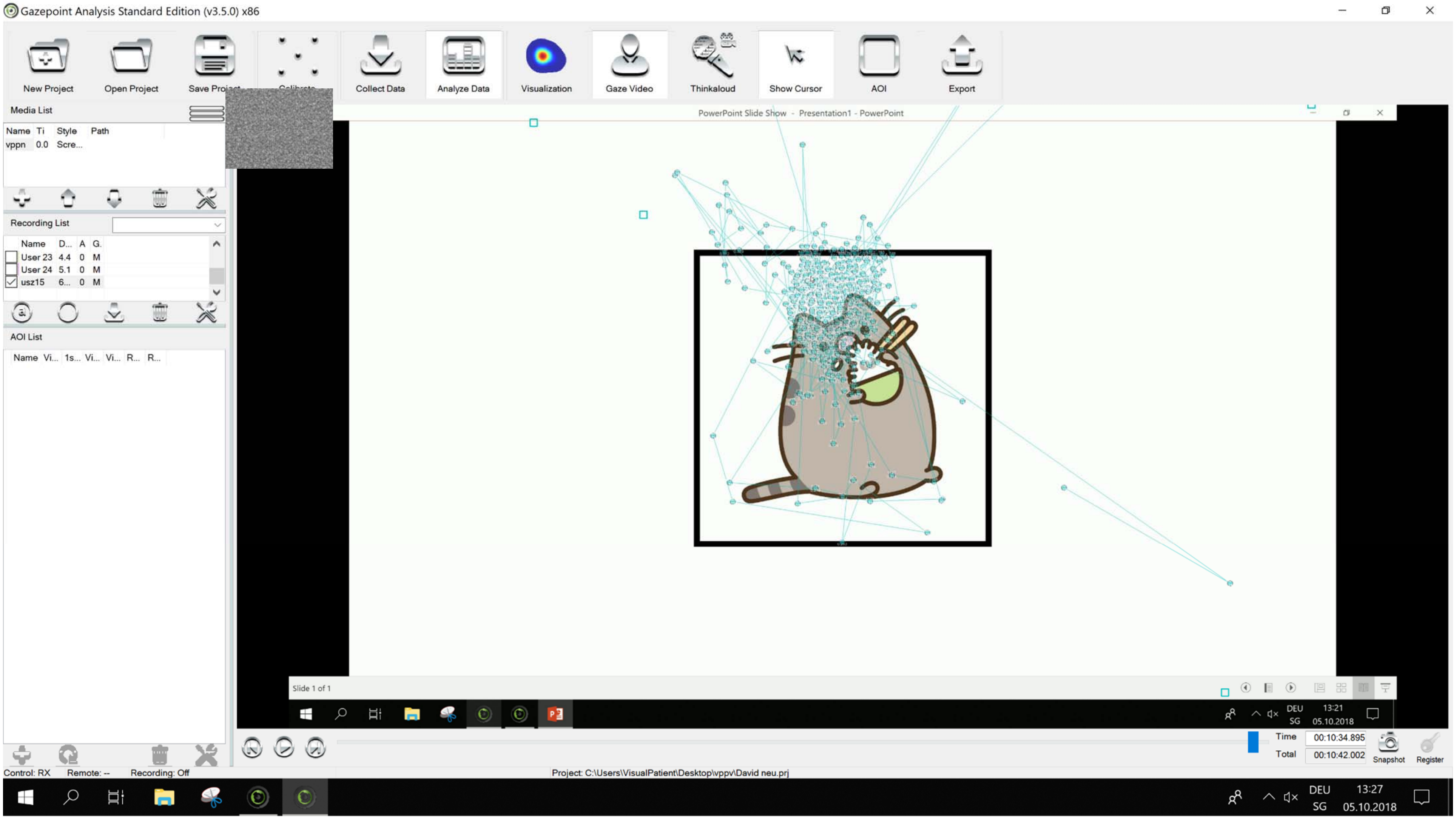

# USZ Szenario 1 (Participant 22)

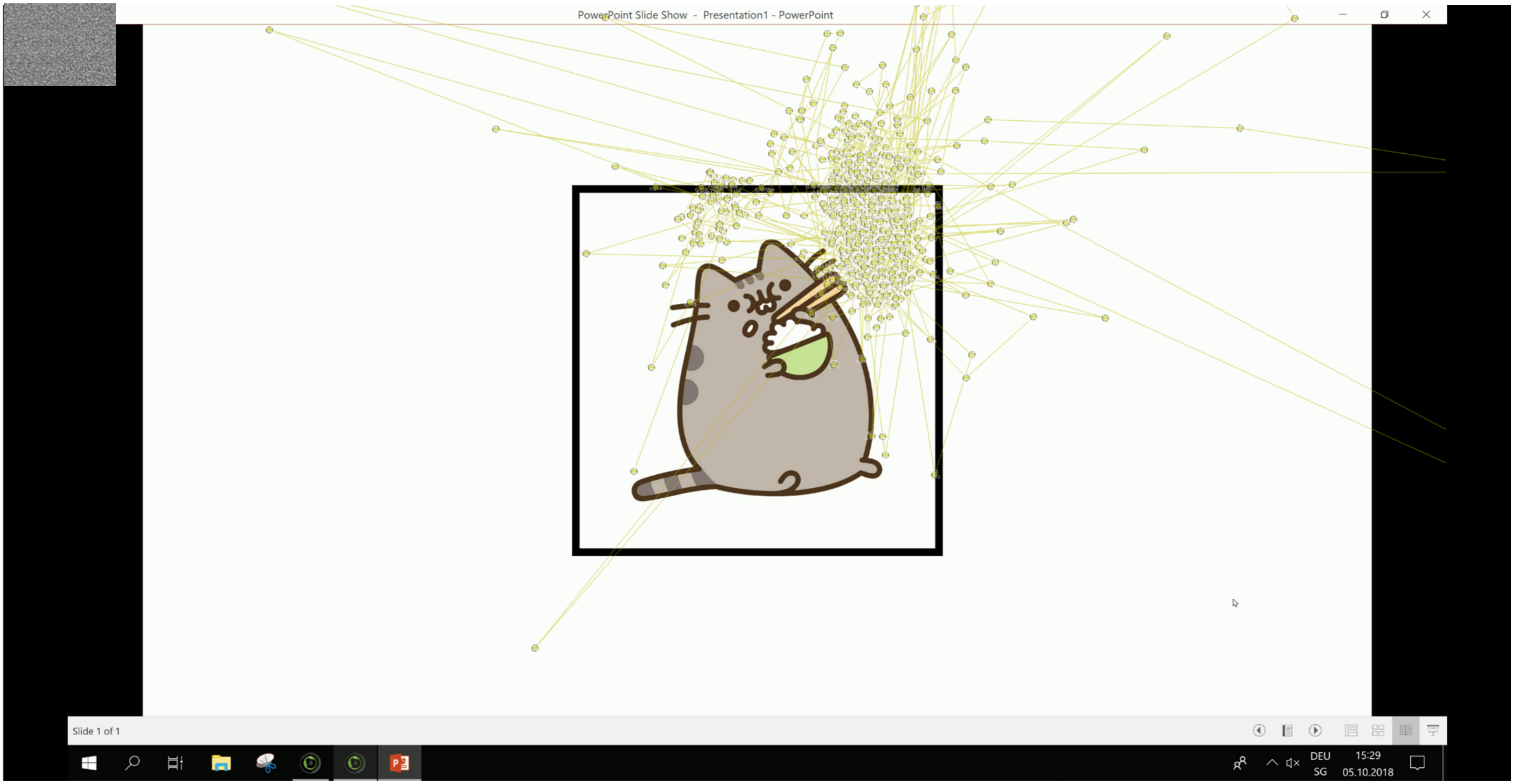

# Gazeplots

# Scenario 2

# KSW Szenario 2 (Participant 23)

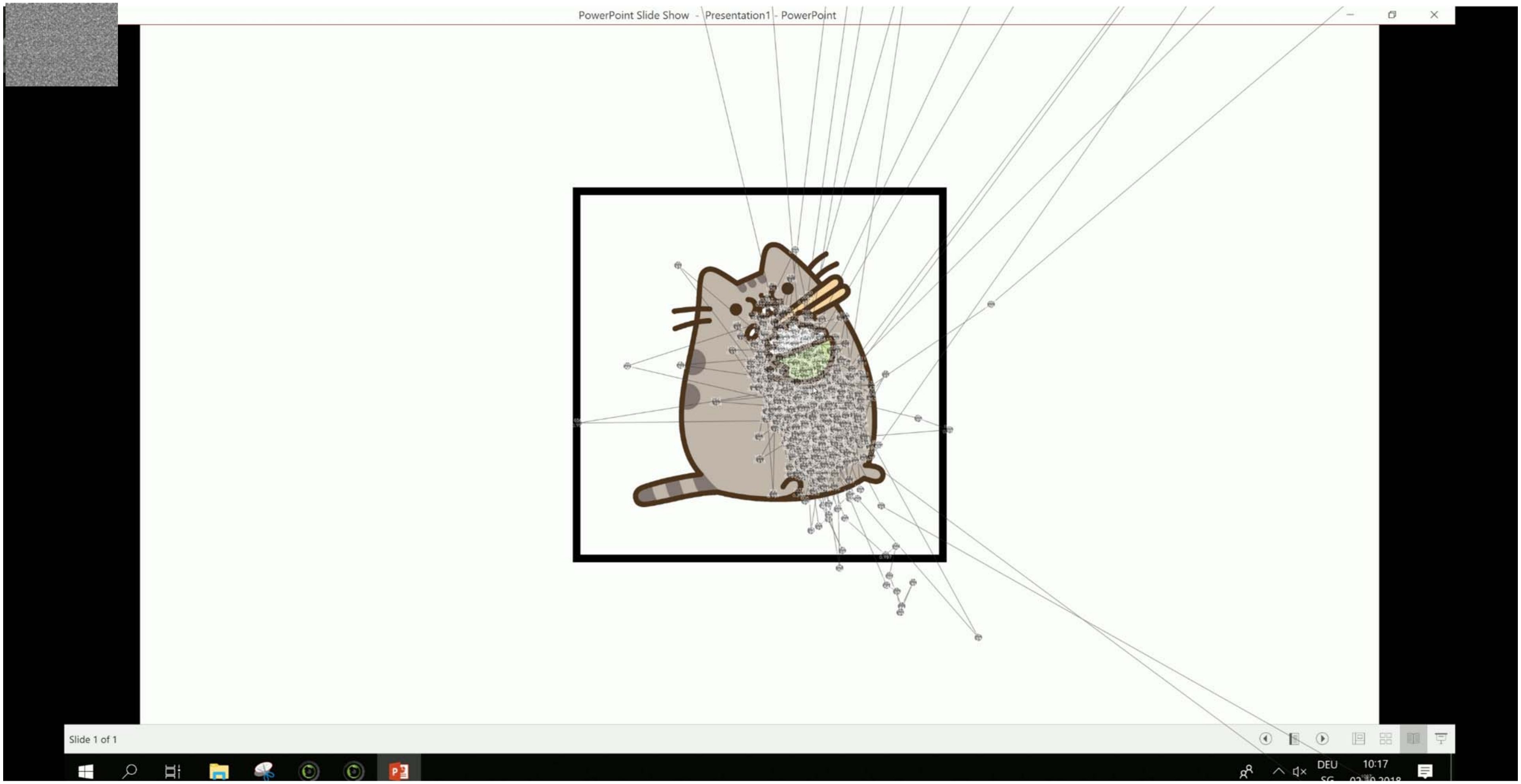

# KSW Szenario 2 (Participant 24)

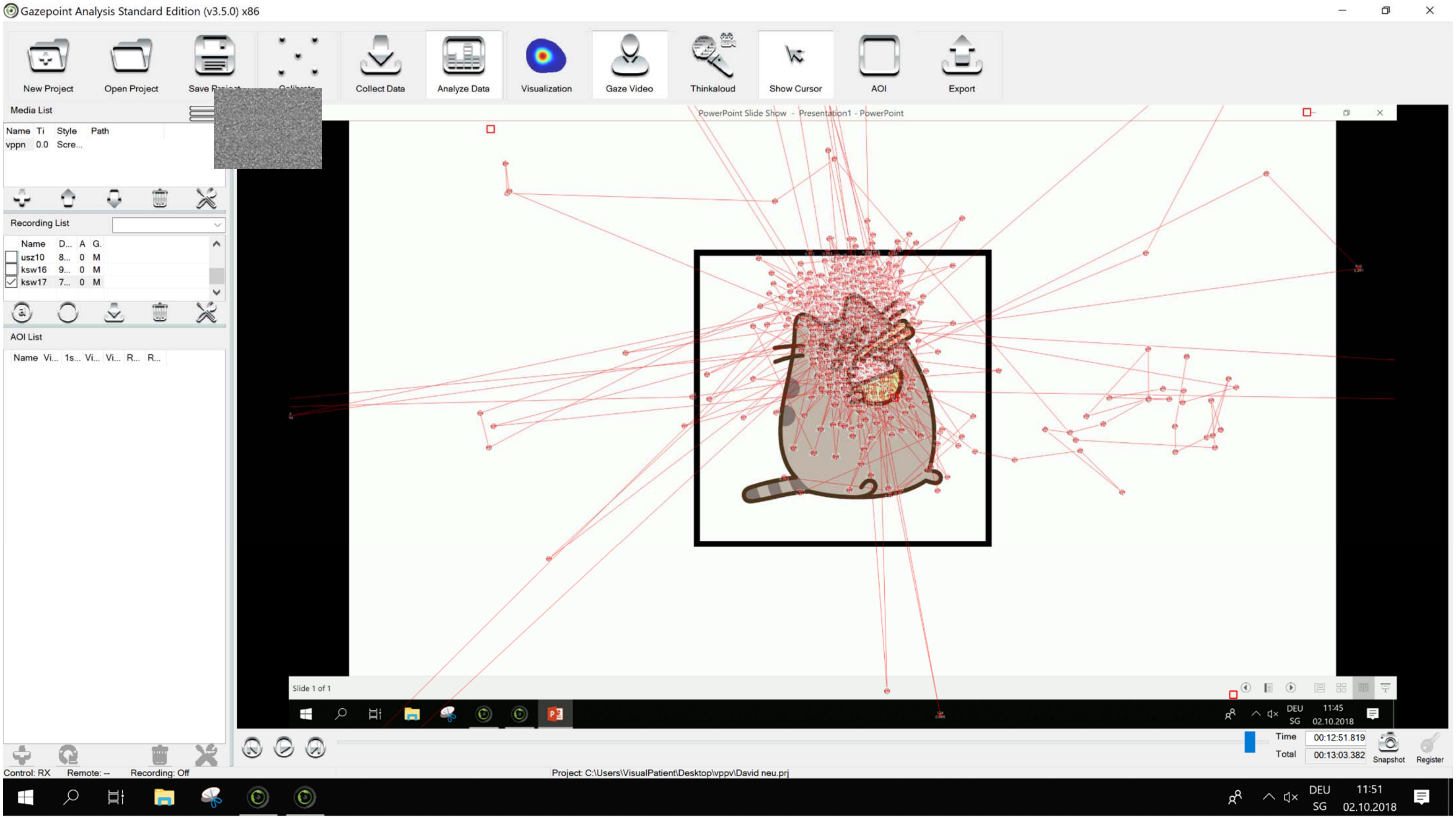

# KSW Szenario 2 (Participant 25)

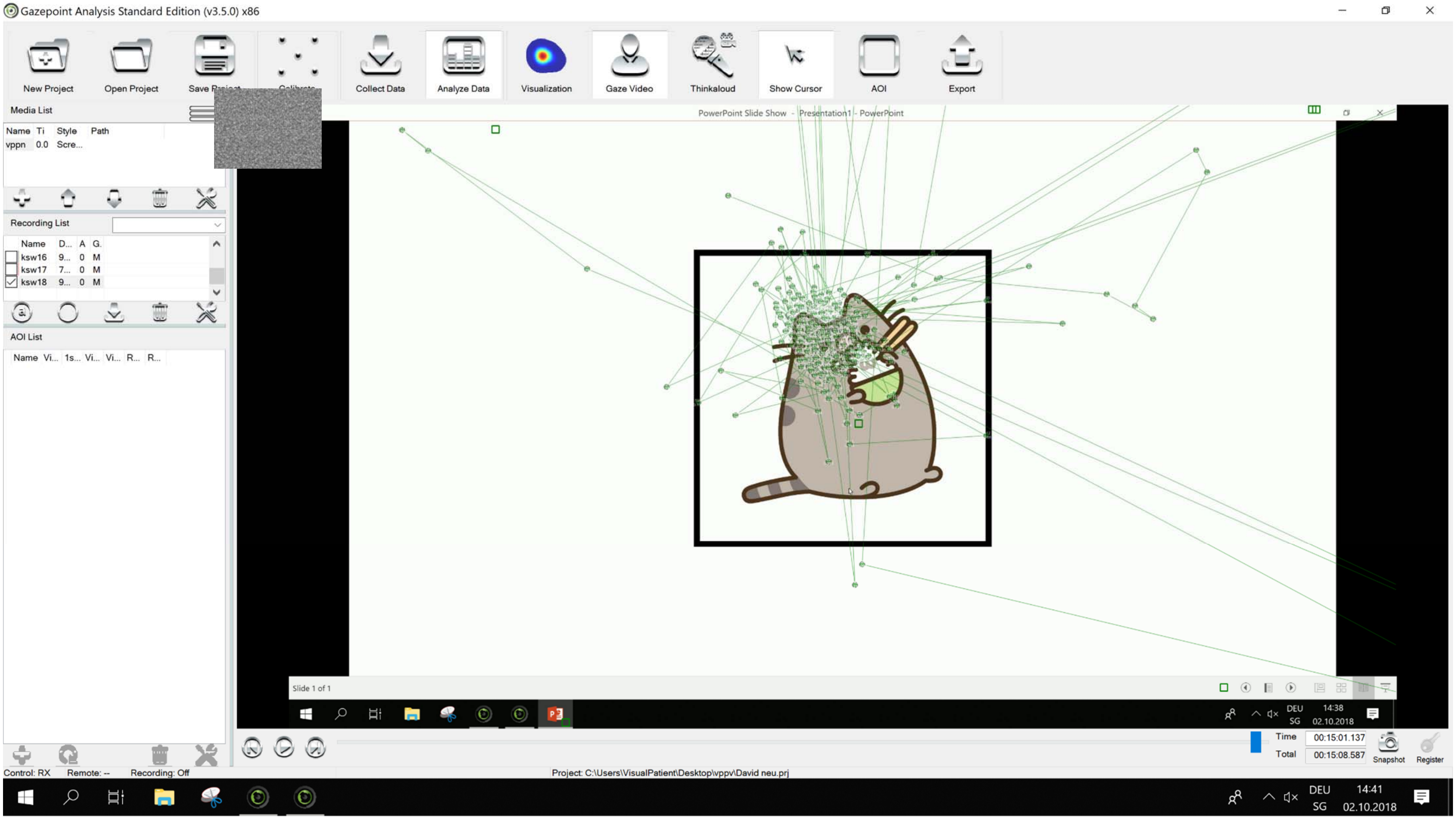

# KSW Szenario 2 (Participant 26)

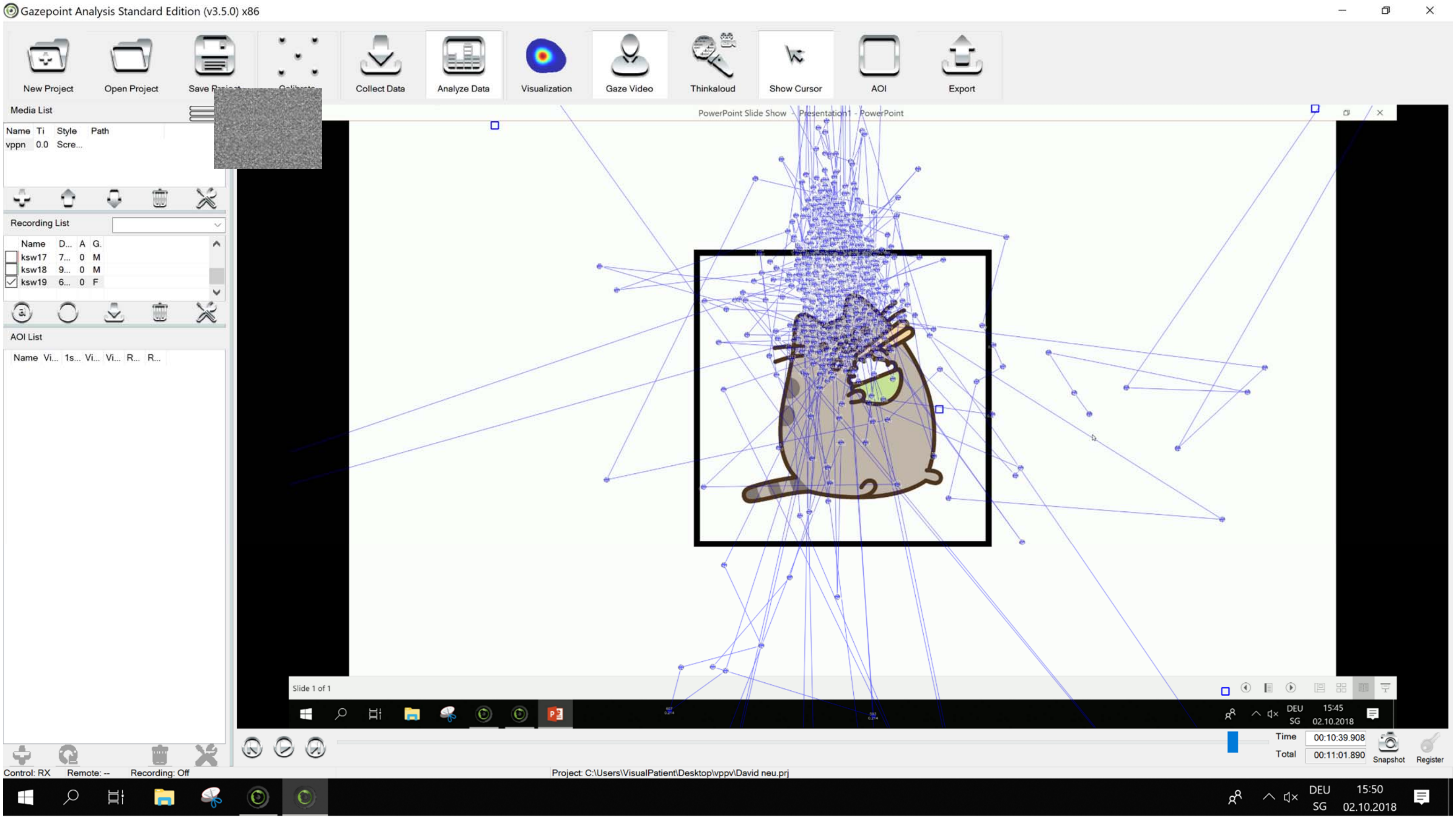

# KSW Szenario 2 (Participant 27)

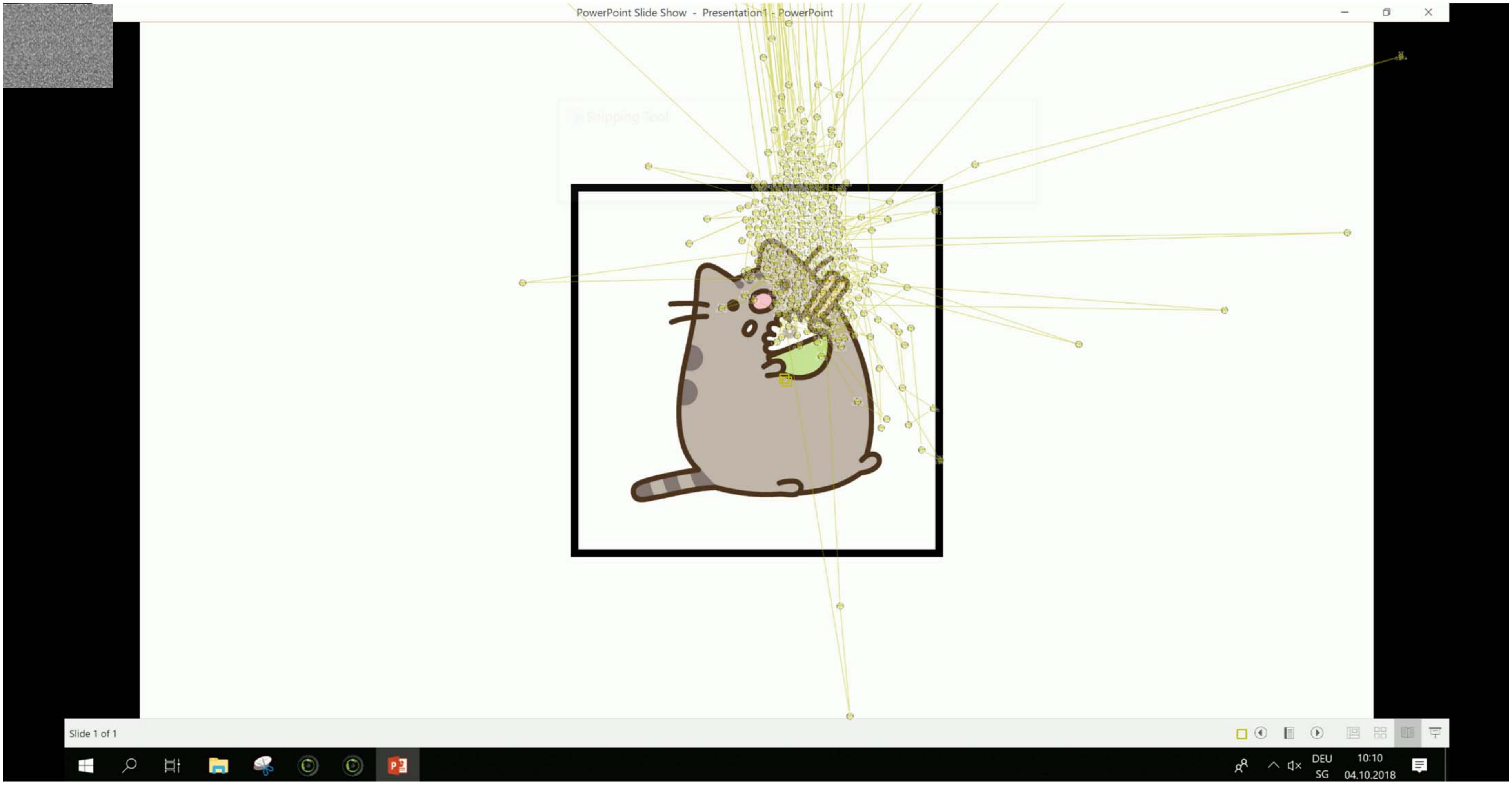

# KSW Szenario 2 (Participant 28)

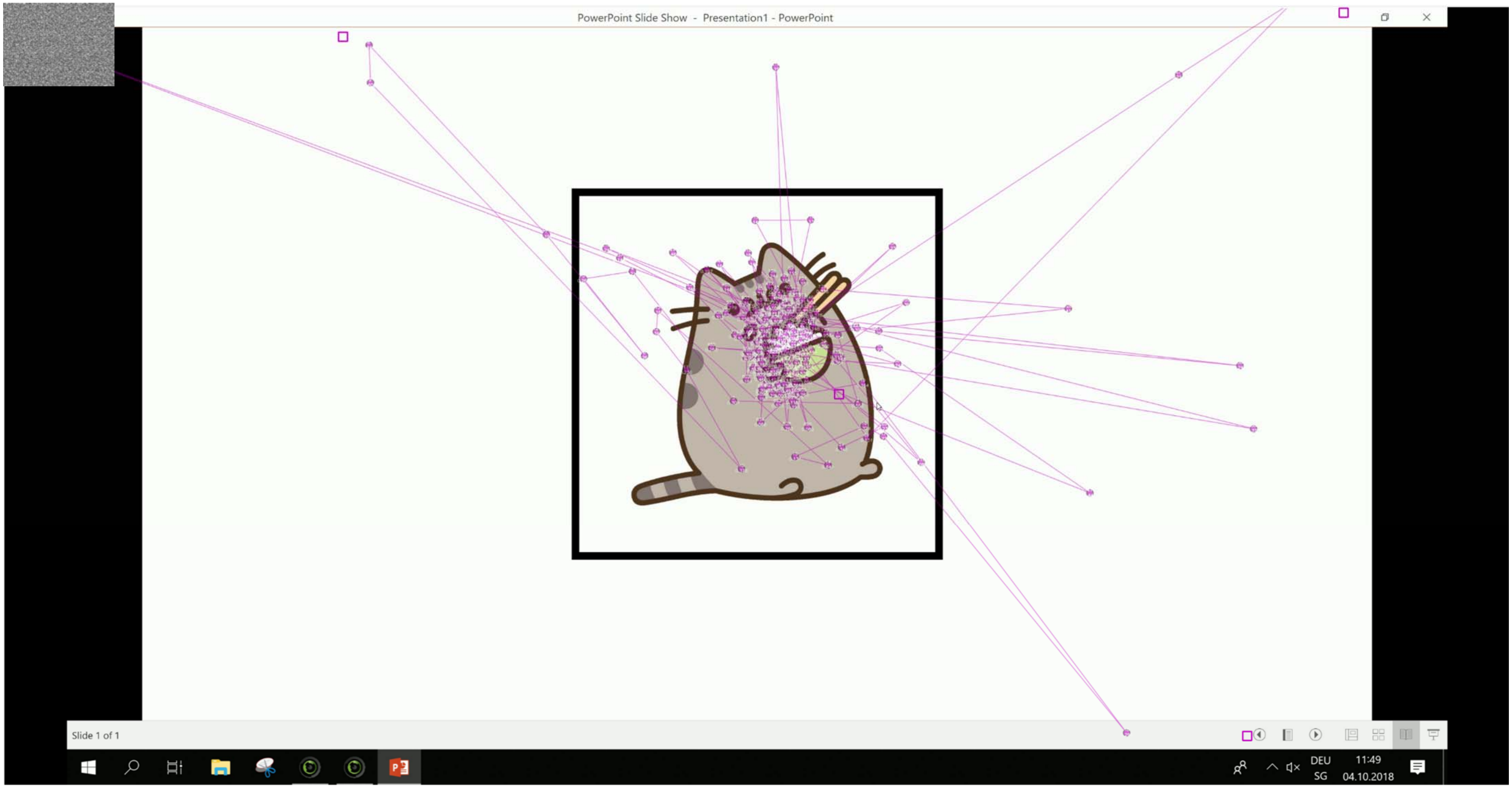

# KSW Szenario 2 (Participant 29)

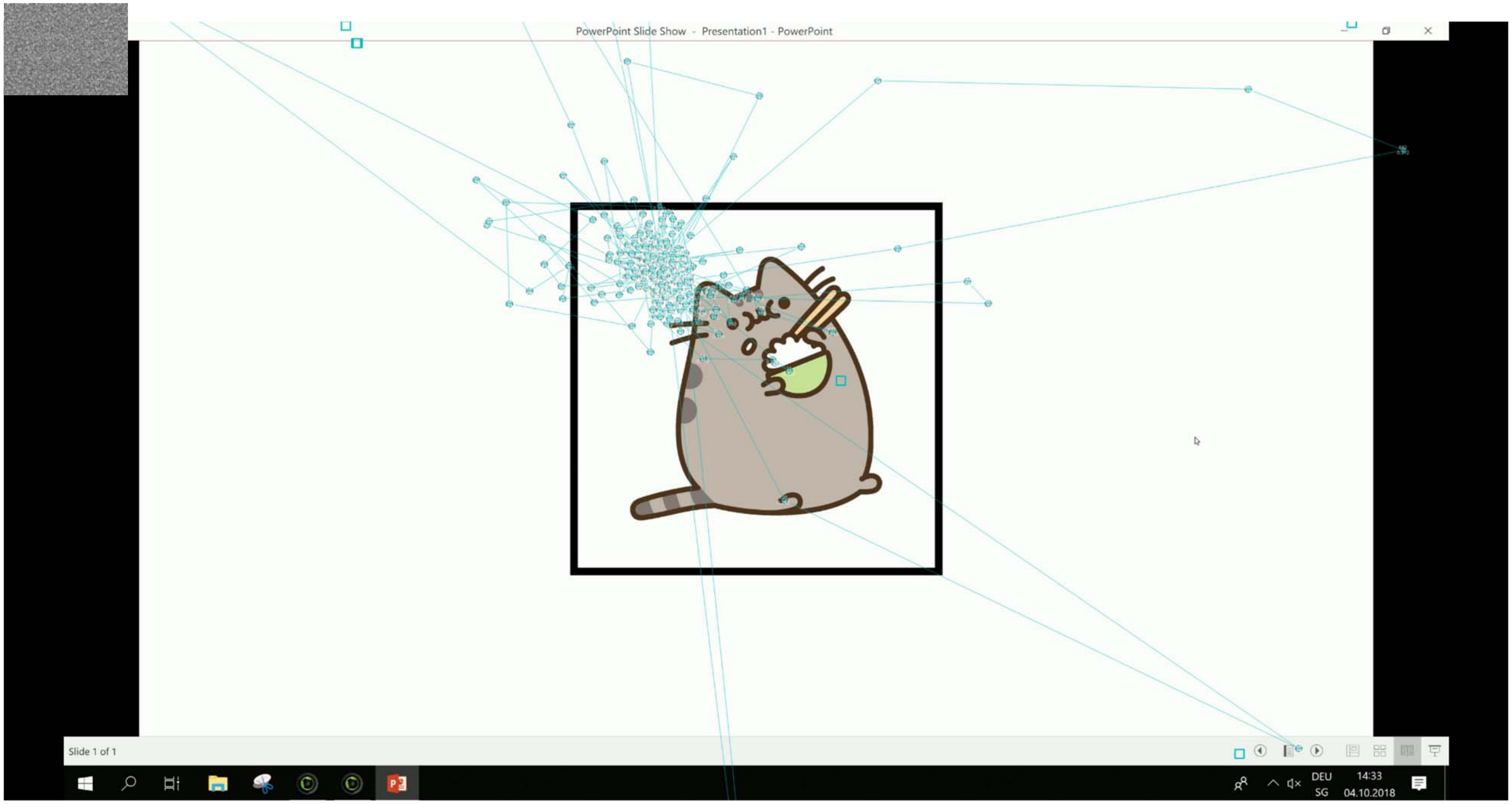

# KSW Szenario 2 (Participant 30)

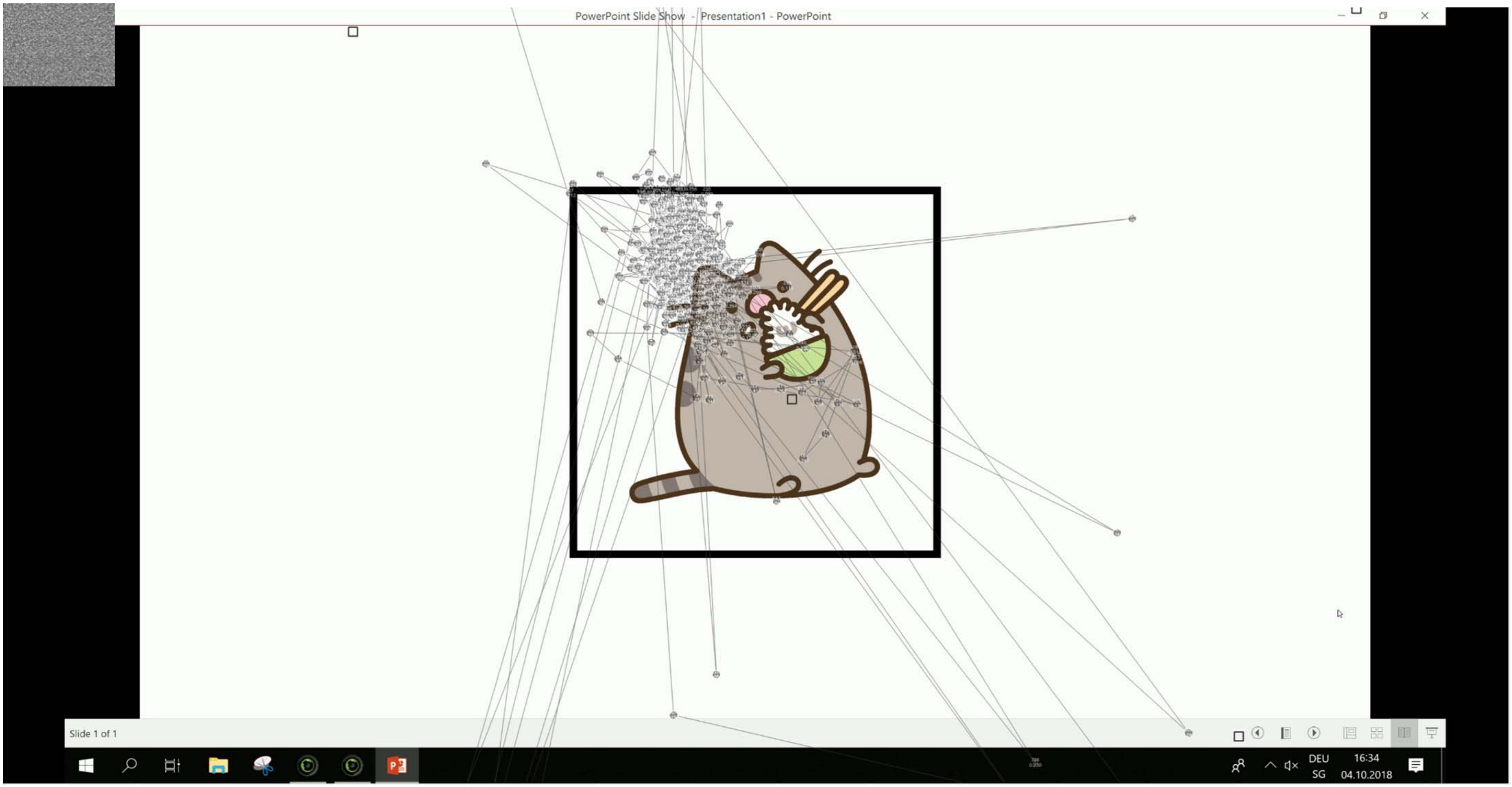

# USZ Szenario 2 (Participant 31)

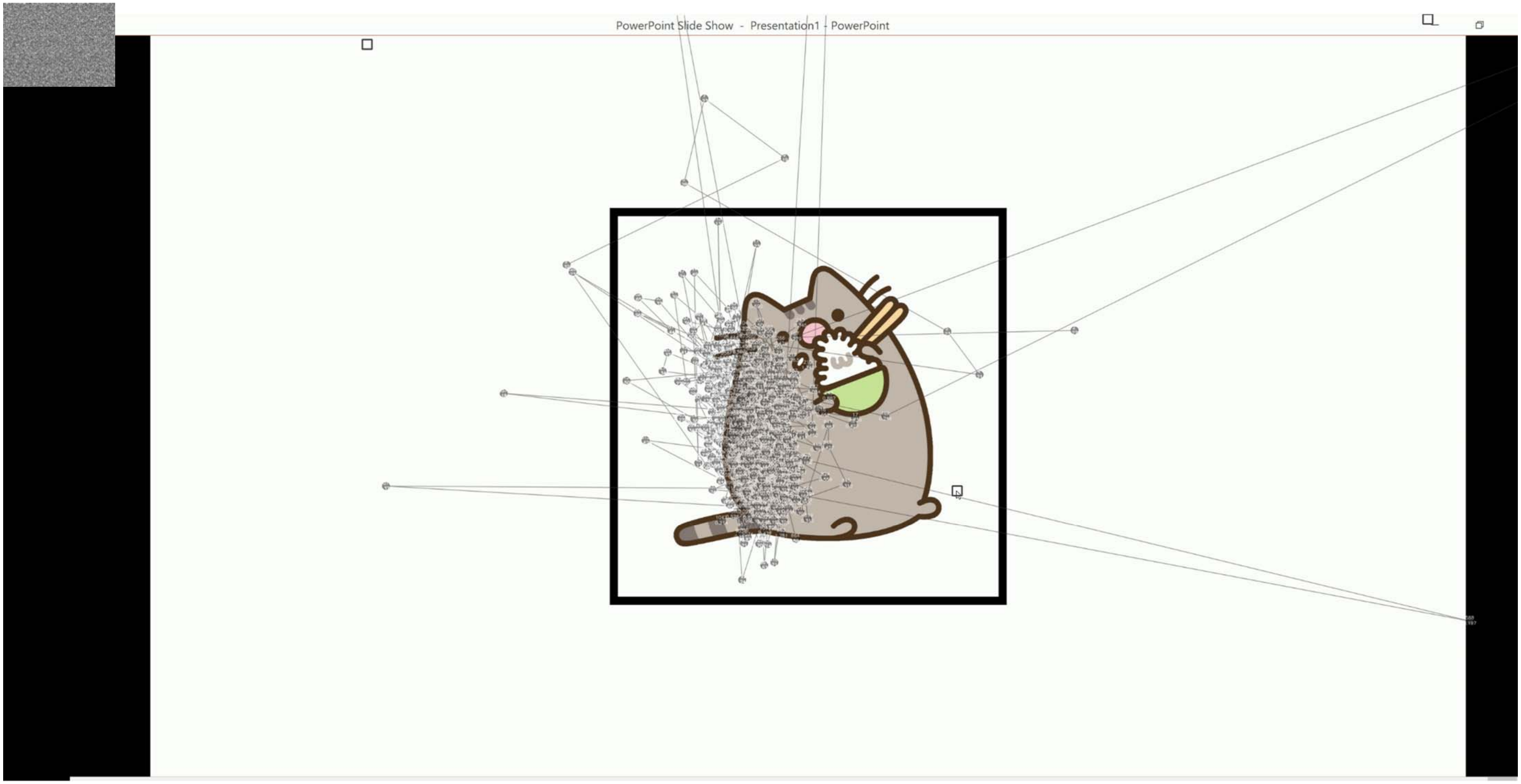

USZ Scenario 2 (Participant 32)

Not included in the data analysis due to view to the left.

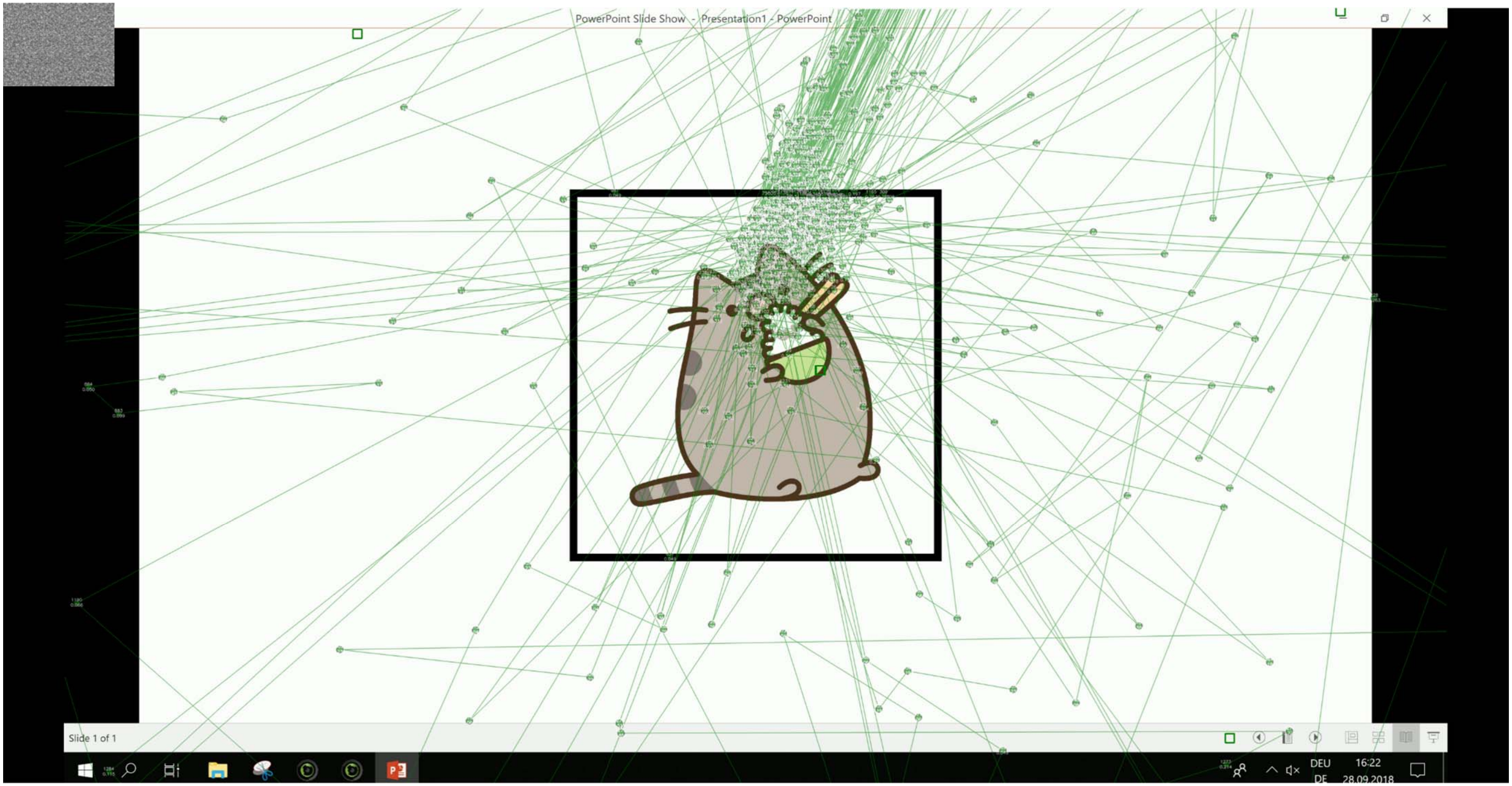

USZ Szenario 2 (Participant 33)

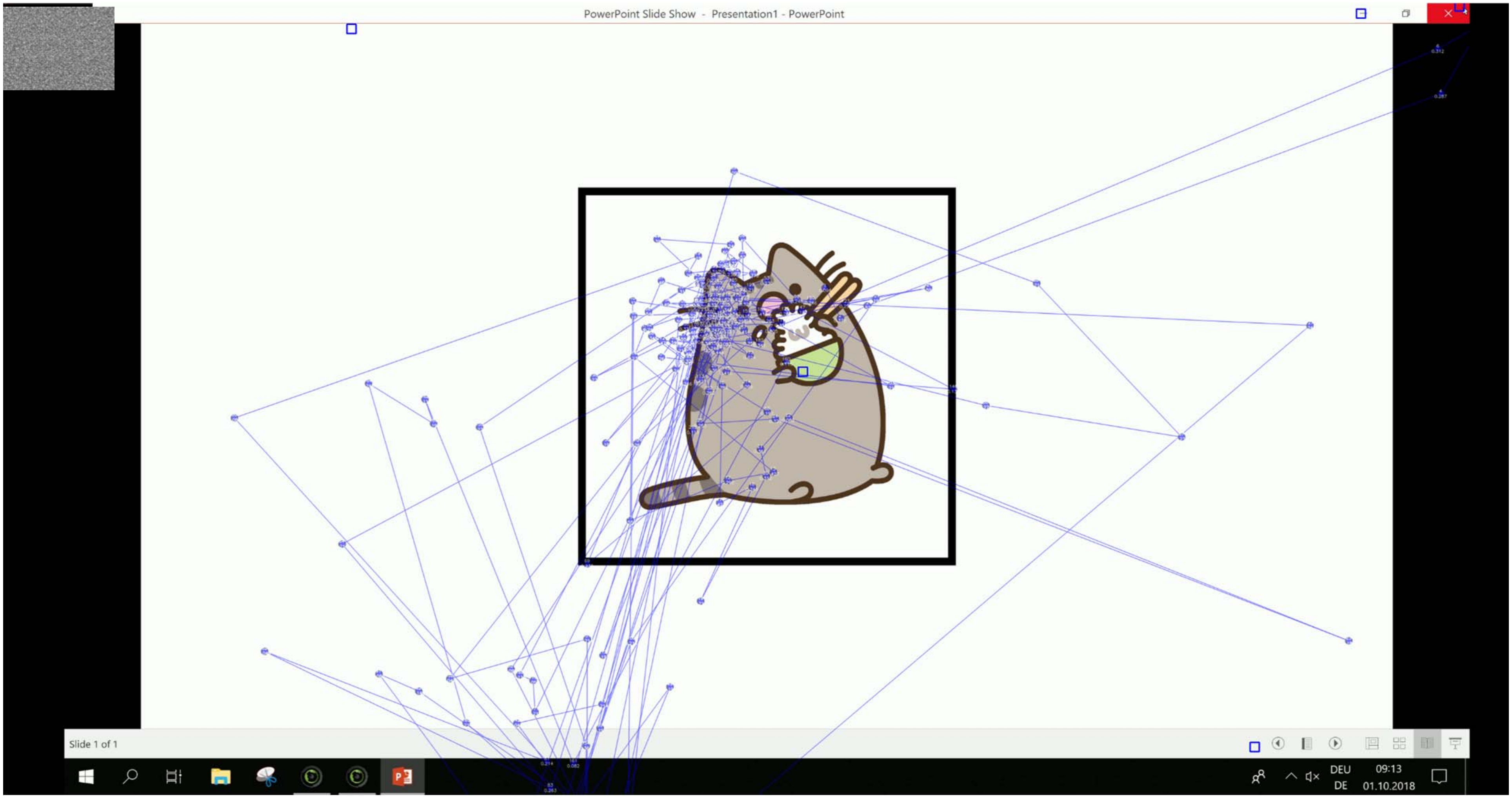

# USZ Szenario 2 (Participant 34)

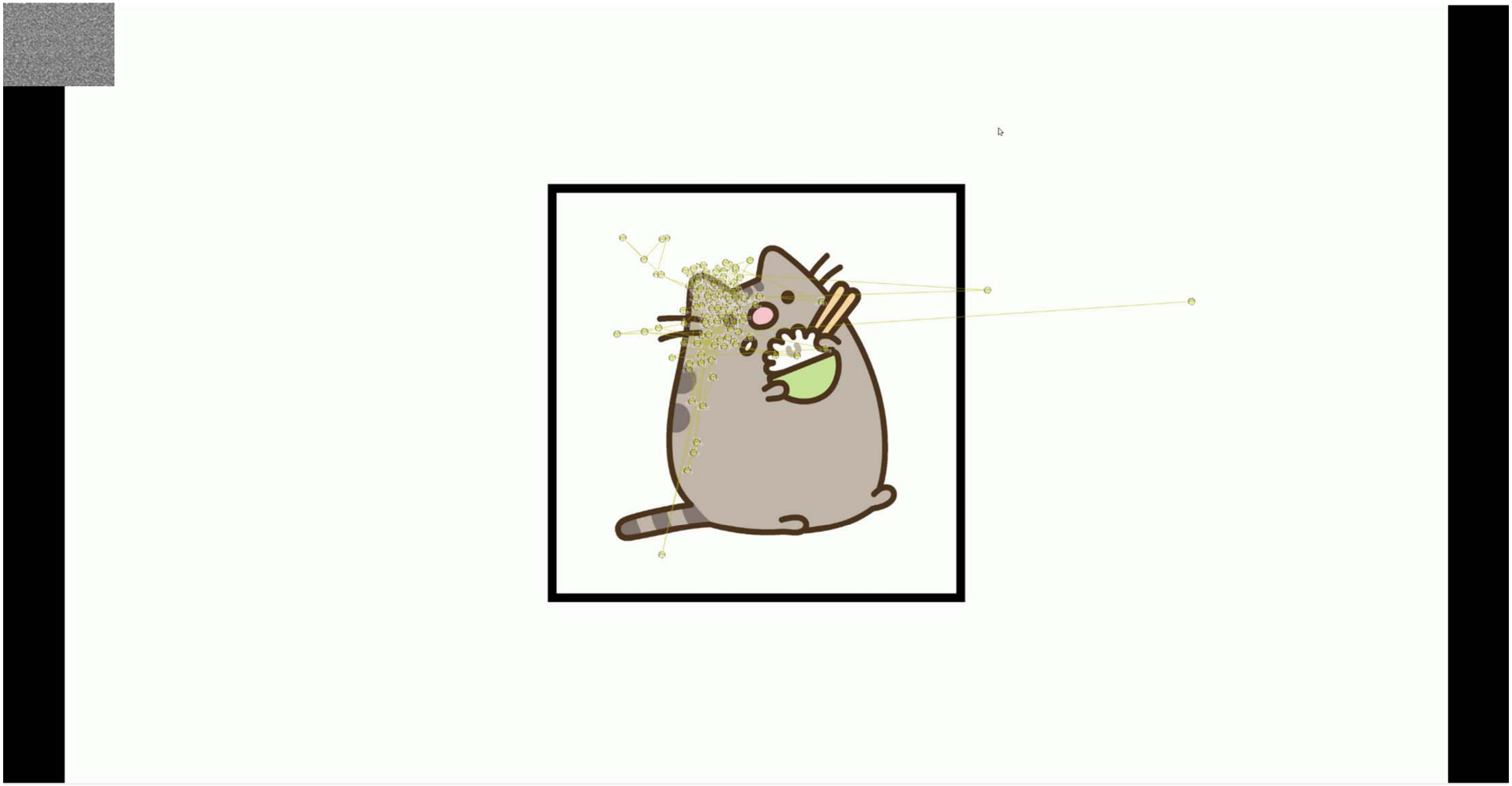

## USZ Szenario 2 (Participant 35)

- No data

# USZ Szenario 2 (Participant 36)

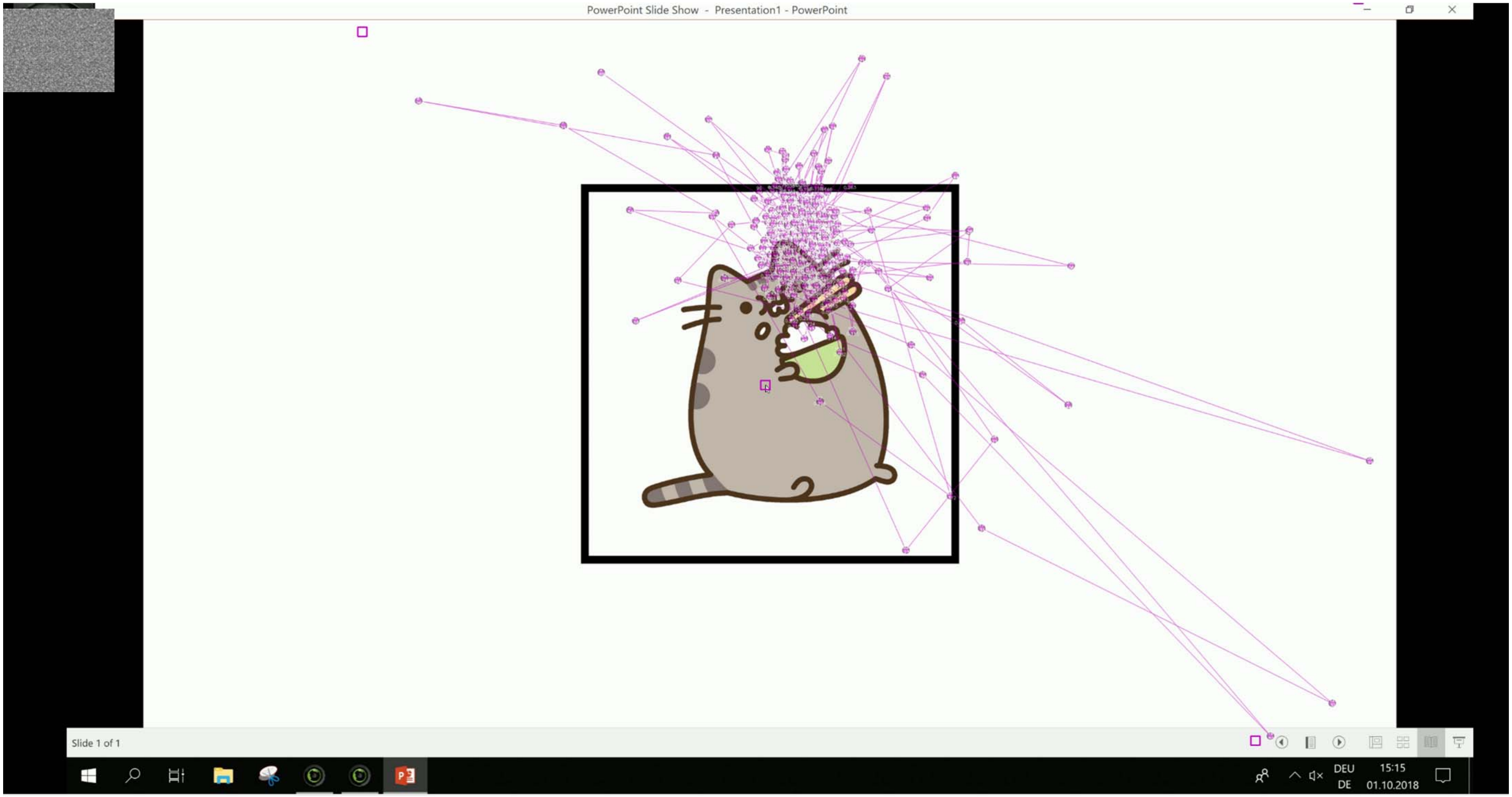

# USZ Szenario 2 (Participant 37)

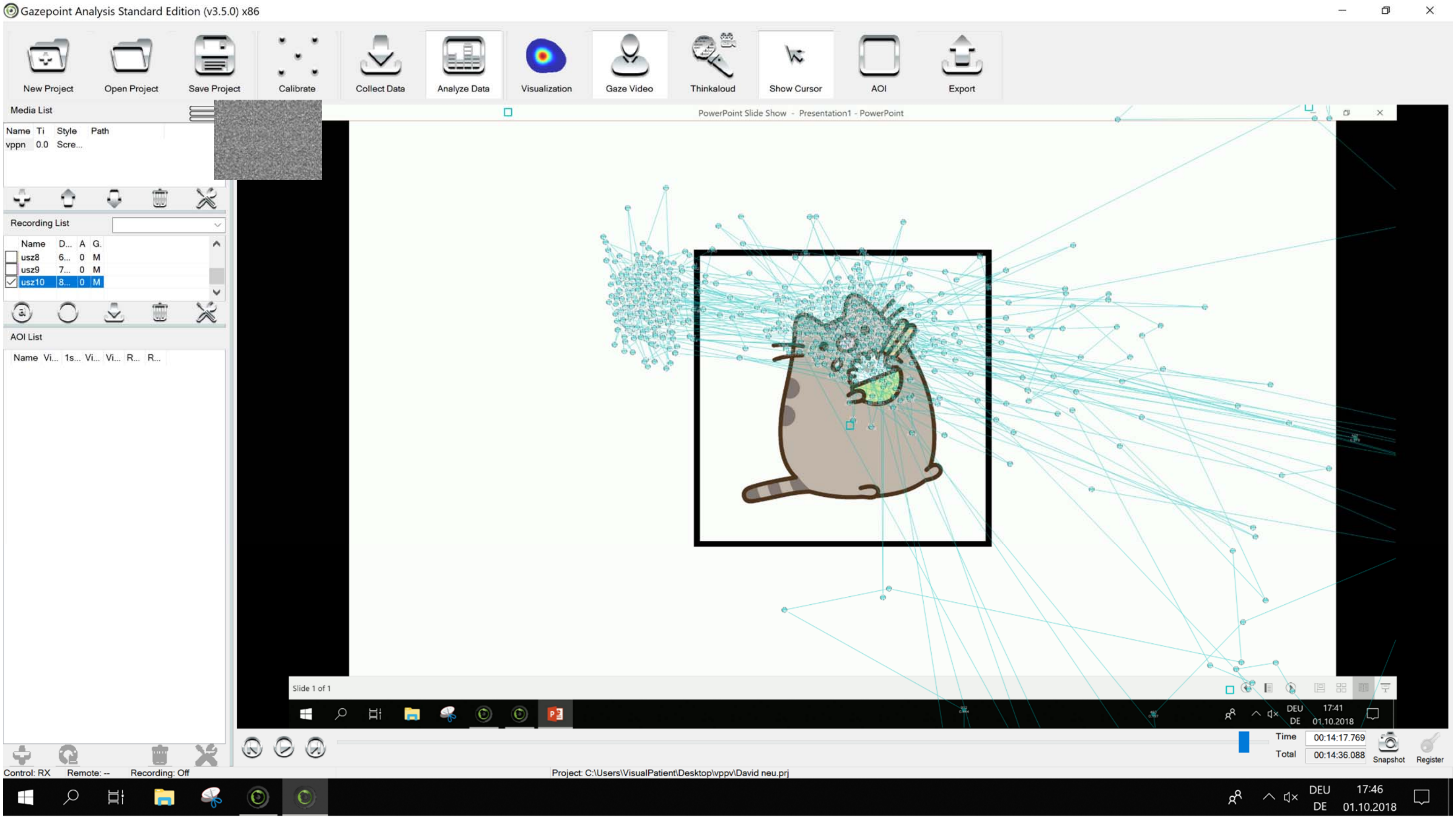

# USZ Szenario 2 (Participant 38)

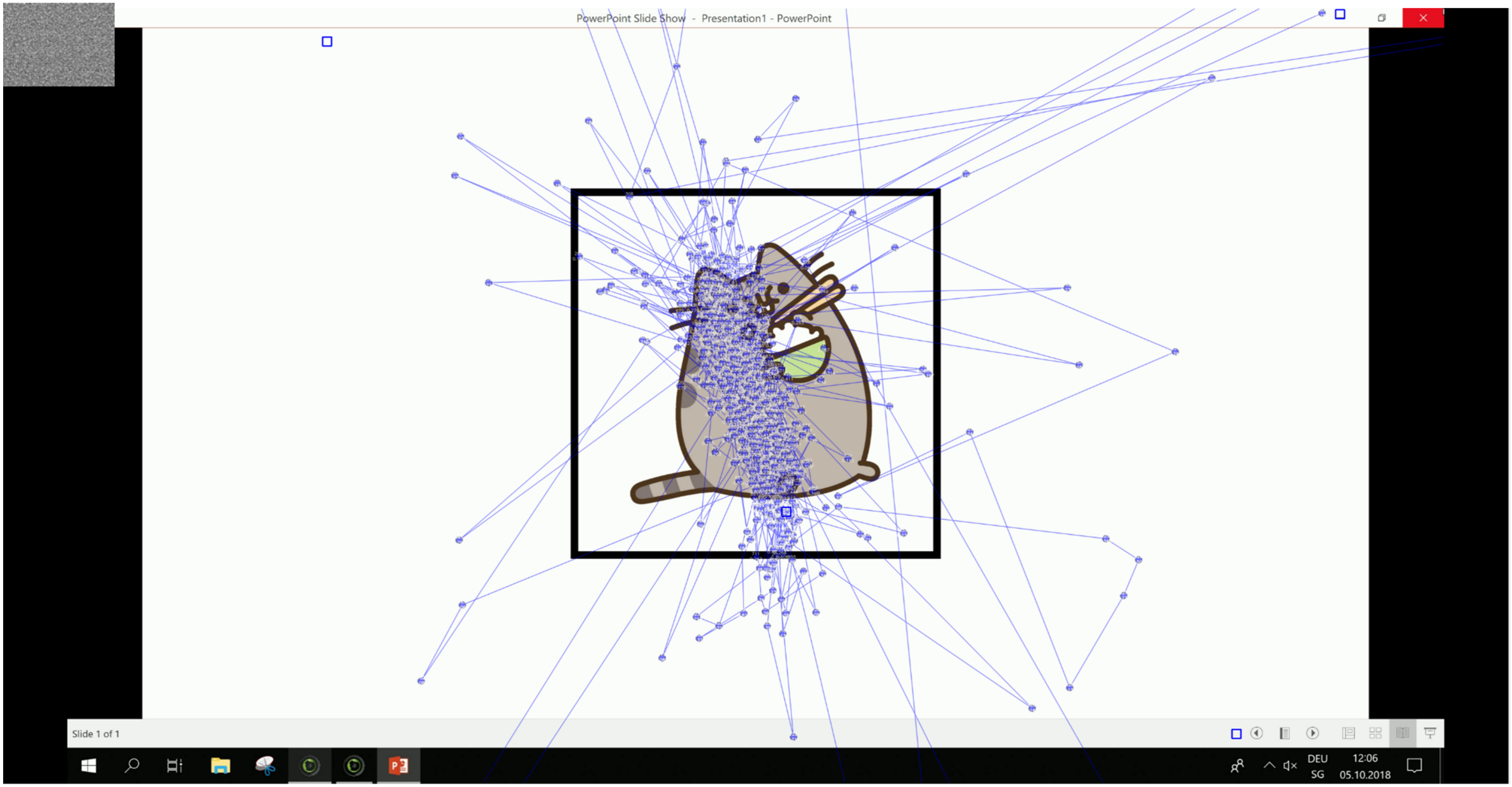

Supplement: Multimedia Appendix 2 [file jmir_v21i7e13041_app2.pdf]
